# LcMYB306 regulates litchi fruit water loss and browning by inhibiting the expression of *LcPIP2;4*

**DOI:** 10.1093/hr/uhaf322

**Published:** 2025-11-21

**Authors:** Xiaoxu Li, Fang Li, Guo Wang, Shujun Wang, Xueren Cao, Ya Wu, Huanling Li, Jiabao Wang

**Affiliations:** National Key Laboratory for Tropical Crop Breeding, Key Laboratory of Integrated Pest Management on Tropical Crops, Ministry of Agriculture and Rural Affairs, Hainan Key Laboratory for Monitoring and Control of Tropical Agricultural Pests, Environment and Plant Protection Institute, Chinese Academy of Tropical Agricultural Sciences, Haikou 571101, China; National Key Laboratory for Tropical Crop Breeding, Key Laboratory of Integrated Pest Management on Tropical Crops, Ministry of Agriculture and Rural Affairs, Hainan Key Laboratory for Monitoring and Control of Tropical Agricultural Pests, Environment and Plant Protection Institute, Chinese Academy of Tropical Agricultural Sciences, Haikou 571101, China; National Key Laboratory for Tropical Crop Breeding, Key Laboratory of Integrated Pest Management on Tropical Crops, Ministry of Agriculture and Rural Affairs, Hainan Key Laboratory for Monitoring and Control of Tropical Agricultural Pests, Environment and Plant Protection Institute, Chinese Academy of Tropical Agricultural Sciences, Haikou 571101, China; National Key Laboratory for Tropical Crop Breeding, Key Laboratory of Integrated Pest Management on Tropical Crops, Ministry of Agriculture and Rural Affairs, Hainan Key Laboratory for Monitoring and Control of Tropical Agricultural Pests, Environment and Plant Protection Institute, Chinese Academy of Tropical Agricultural Sciences, Haikou 571101, China; National Key Laboratory for Tropical Crop Breeding, Key Laboratory of Integrated Pest Management on Tropical Crops, Ministry of Agriculture and Rural Affairs, Hainan Key Laboratory for Monitoring and Control of Tropical Agricultural Pests, Environment and Plant Protection Institute, Chinese Academy of Tropical Agricultural Sciences, Haikou 571101, China; National Key Laboratory for Tropical Crop Breeding, Key Laboratory of Integrated Pest Management on Tropical Crops, Ministry of Agriculture and Rural Affairs, Hainan Key Laboratory for Monitoring and Control of Tropical Agricultural Pests, Environment and Plant Protection Institute, Chinese Academy of Tropical Agricultural Sciences, Haikou 571101, China; National Key Laboratory for Tropical Crop Breeding, Key Laboratory of Integrated Pest Management on Tropical Crops, Ministry of Agriculture and Rural Affairs, Hainan Key Laboratory for Monitoring and Control of Tropical Agricultural Pests, Environment and Plant Protection Institute, Chinese Academy of Tropical Agricultural Sciences, Haikou 571101, China; National Key Laboratory for Tropical Crop Breeding, Key Laboratory of Integrated Pest Management on Tropical Crops, Ministry of Agriculture and Rural Affairs, Hainan Key Laboratory for Monitoring and Control of Tropical Agricultural Pests, Environment and Plant Protection Institute, Chinese Academy of Tropical Agricultural Sciences, Haikou 571101, China

## Abstract

Pericarp browning of postharvest litchi is a significant obstacle to the industry’s high-quality development. Water loss from the pericarp is a key factor triggering browning, but the regulatory mechanism of water metabolism and its relationship with browning remain unclear. In this study, we found that aquaporin activity inhibitors (HgCl_2_) can delay both water loss and browning in litchi. LcPIP2;4, a plasma membrane intrinsic protein (PIP) family member exhibiting high expression in the litchi pericarp and the greatest water transport activity, is significantly downregulated during water loss and browning. Further analysis revealed that HgCl₂ suppresses both the expression and water transport activity of LcPIP2;4, indicating a close association with the observed browning phenotype. By constructing transient overexpression fruits and transgenic callus tissues of *LcPIP2;4* and measuring the water loss rate and browning index, we confirmed that LcPIP2;4 positively regulates water loss and browning in litchi. Through weighted gene co-expression network, LcPIP2;4 promoter sequence, and Quantitative Reverse Transcription Polymerase Chain Reaction (qRT-PCR) analysis, we identified 10 potential interacting transcription factors. Yeast one-hybrid, dual-luciferase reporter assay, chromatin immunoprecipitation analysis, and electrophoretic mobility shift assay confirmed that LcMYB306 specifically binds to the *LcPIP2;4* promoter. In LcMYB306 overexpressing fruits and embryogenic callus, *LcPIP2;4* expression was suppressed, resulting in delayed water loss and browning. In contrast, in CRISPR/Cas9-edited *LcMYB306* callus, *LcPIP2;4* expression was upregulated, and water loss and browning were accelerated, confirming that LcMYB306 negatively regulates this process. This study demonstrates that LcMYB306 delays postharvest water loss and browning in litchi by repressing LcPIP2;4 transcriptionally expression. It provides a theoretical foundation and key target gene for developing litchi varieties resistant to browning.

## Introduction

Litchi (*Litchi chinensis* Sonn.) is native to tropical and subtropical regions and is highly valued by consumers for its rich nutritional content, vibrant color, distinctive fragrance, and sweet taste [1–3]. Postharvest browning severely restricts the fruit's storage, transportation, and market value, leading to an annual loss of 20% to 30%, thus posing a significant bottleneck in industry development [4, 5]. Current research primarily focuses on oxidative stress and membrane damage, anthocyanin metabolic imbalance, energy metabolism, key enzymes involved in browning, and the development of postharvest treatment technologies [6–10]. Notably, the obstruction of water transport between the fruit pericarp and flesh, which results in rapid water loss from the pericarp, is a key factor in browning [4, 5, 11].

Several studies have shown that inhibiting postharvest water loss can effectively delay browning [4, 11, 12]. Existing research on water loss inhibition in litchi mainly focuses on postharvest technologies such as packaging, coating films, and low-temperature storage [13–16]. In previous studies, we found that bagging treatments significantly delayed browning by reducing the water loss rate of the fruit peel [17]. Recent studies have shown that within 48 hours postharvest, the rate of browning correlates positively with the amount of water loss, and water loss can trigger a wax metabolism response [5]. Additionally, supplying water through the flower stalk can effectively maintain the peel's water content and suppress browning [11]. However, current methods face challenges, such as high costs and limited effectiveness [11]. For example, packaging may lead to condensation and microbial growth, coatings may fail to cover the entire peel, and cold storage can result in chilling injury while being costly. Therefore, exploring the genes involved in water loss regulation in litchi and elucidating their mechanisms, followed by molecular breeding to develop water loss-resistant varieties, is the key to overcoming these technological bottlenecks. However, research in this area is still in its early stages, primarily focusing on gene screening, with insufficient functional verification and lack of a systematic mechanism analysis. Existing studies suggest that cuticular metabolism genes *LcCER1* and *LcKCS1* may be involved in regulating water loss from the peel [5], but direct evidence is still lacking.

Aquaporins (AQPs), located on the plasma membrane or organelle membranes, serve as transport channels for substances, with H_2_O being their primary substrate [18, 19]. Plasma membrane intrinsic protein (PIPs), located on the cytoplasmic membrane, mediate the transport of H_2_O in and out of cells, thereby regulating cellular water balance and influencing various physiological processes [20, 21]. Plasma membrane intrinsic proteins are divided into two subfamilies: PIP1 and PIP2, with PIP2 exhibiting higher water channel activity than PIP1 family members [22, 23]. In postharvest fruits, the peel serves as the first barrier to water loss and may delay this process by downregulating PIP gene expression. For instance, the reduction in PIP abundance in pear peel significantly enhances water retention [24], and decreased PIP expression in citrus postharvest has also been shown to reduce water loss [25]. Studies have demonstrated that R2R3-MYB transcription factors can regulate the transcription of PIPs in postharvest fruits. For example, the citrus MYB96 reduces water transport by inhibiting AQP expression, thereby lowering the fruit’s water loss rate [25]. In our previous studies, we observed that several PIPs in litchi exhibited a downregulation pattern similar to that of citrus during postharvest storage [17], suggesting a potentially similar transcriptional regulation mechanism.

This study confirms the role of LcPIP2;4 in regulating water loss and browning processes in litchi through transient expression and transgenic callus technology. Based on weighted gene co-expression network analysis and promoter interaction verification, we identified the transcription factor LcMYB306, which specifically bind with the *LcPIP2;4* promoter. Both transient expression and transgenic callus experiments demonstrated that MYB306 reduces tissue water loss and browning by inhibiting *LcPIP2;4* expression. These findings reveal the ‘LcMYB306-LcPIP2;4’ regulatory pathway involved in mediating water loss and browning in litchi, offering a new perspective for studying postharvest fruit water regulation mechanisms.

## Results

### Aquaporin inhibitors HgCl_2_ inhibit water loss and browning of litchi fruit

**Figure 1:**
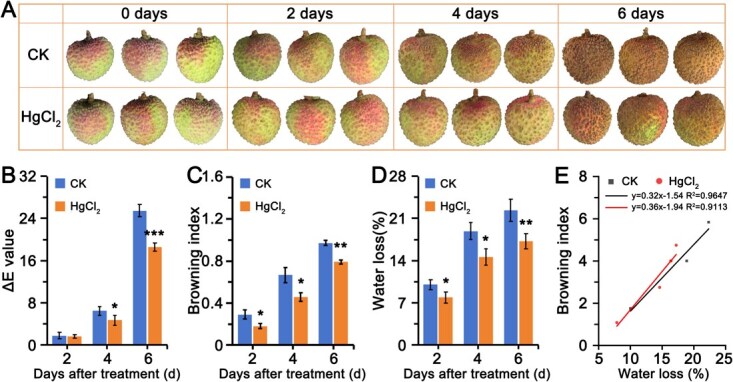
Changes in browning and water loss in litchi following 200 μM HgCl_2_ treatment over a 6-day storage period at room temperature. (A) Phenotypic observations, (B) chromatism value, (C) browning index, (D) water loss, and (E) correlation analysis of browning index and water loss of litchi pericarps after HgCl_2_ treatment over 6 days of storage. Means and standard errors were calculated from three biological replicates, each treatment consisting of 30 fruits. Asterisks (^*^, ^**^, and ^***^) indicate significant differences at *P* < 0.05, *P* < 0.01, and *P* < 0.001, respectively (*t*-test).

HgCl₂, a specific inhibitor of aquaporins, exerts its effect by binding to cysteine residues near the water channel pores of aquaporins, inducing a conformational change in the protein and thereby inhibiting water transport [26–28]. To investigate the impact of aquaporin activity on water loss and browning in litchi fruit, this study employed HgCl₂ (0.2 mM) to treat the fruit. In the litchi fruit experiment, HgCl₂ treatment significantly delayed the browning process compared to the control group (Fig. 1A). On the fourth and sixth days of post-harvest storage, the chromatic values for the HgCl₂-treated group were 4.68 and 18.57, respectively, whereas the control group exhibited values of 6.43 and 25.49. The chromatic value in the treated group was significantly lower than that of the control group (Fig. 1B). Furthermore, during storage, both the browning index and water loss rate were significantly reduced in the HgCl₂-treated group compared to the control group. Specifically, the browning index for the treated group was 61.90% (2 days), 68.75% (4 days), and 81.43% (6 days) of the control group, while the water loss rate was 78.36% (2 days), 77.34% (4 days), and 77.05% (6 days) of the control group (Fig. 1C and D). Correlation analysis revealed a positive relationship between the browning index and water loss (Fig. 1E), indicating that increased water loss is associated with more pronounced browning. Together, these results suggest that the water transport activity of aquaporins may regulate water loss and the browning process in litchi fruit.

### Aquaporin inhibitors HgCl_2_ suppresses both the expression of LcPIP2;4 and its water transport activity

Cell membrane-localized aquaporin known as PIPs play a crucial role in regulating postharvest water metabolism in fruits [24, 25]. We screened the ‘Feizixiao’ litchi genome assembly published by Hu *et al*. [1]. BLASTP was performed using AtPIP and OsPIP protein sequences as queries, alongside HMM conserved domain (PF00230) and domain integrity analysis, resulting in the identification of nine LcPIPs (Table S2). Based on the classification system of AtPIPs and OsPIPs, a phylogenetic tree constructed by the neighbor-joining method, which grouped these genes into two subfamilies: LcPIP1 (four members) and LcPIP2 (five members) (Fig. S1). These genes were designated *LcPIP1;1*-*LcPIP1;4* and *LcPIP2;1*-*LcPIP2;5*.

**Figure 2:**
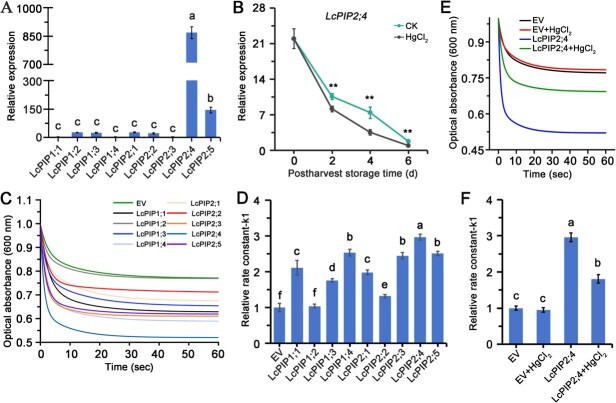
Effect of aquaporin inhibitors HgCl_2_ on the expression and water transport capacity of LcPIP2;4. (A) Relative expression of *LcPIPs* in litchi pericarps. (B) Relative expression of *LcPIP2;4* in litchi pericarps following HgCl_2_ treatment during postharvest storage at room temperature. Asterisks (* and **) indicate significant differences at *P* < 0.05 and *P* < 0.01, respectively (*t*-test). (C) Water transport measurement of LcPIPs protein in *saccharomyces cerevisiae*. LcPIP1;1, LcPIP1;2, LcPIP1;3, LcPIP1;4, LcPIP2;1, LcPIP2;2, LcPIP2;3, LcPIP2;4 and LcPIP2;5 was expressed in yeast. Spheroplasts were prepared from these cell lines and treated with hypo-osmotic shock. Rupture of the spheroplasts due to water influx was monitored by measuring the decrease in OD_600_ nm every 2 seconds. The optical absorbance was expressed as a relative value with the starting OD_600_ nm value as 1.0. (D) Relative rate constant values-k1 for the absorbance-time fitted curves of different LcPIPs expressed in yeast spheroplasts. Data from six independent experiments and fitted to two-phase exponential decay functions. Lowercase letters indicated significant differences among the data based on one-way ANOVA and Tukey’s multiple range tests (*P* < 0.05). (E) Effect of HgCl_2_ treatment to optical absorbance of LcPIP2;4 yeast spheroplasts. LcPIP2;4 was expressed in yeast and spheroplasts were prepared from these cell lines and treated with hypo-osmotic shock. In the inhibitor treatment group, protoplasts were pre-incubated in 200 μM HgCl₂-containing assay buffer for 20 minutes. Rupture of the spheroplasts due to water influx was monitored by measuring the decrease in OD_600_ nm every 2 seconds. The optical absorbance was expressed as a relative value with the starting OD_600_ nm value as 1.0. (F) Effect of HgCl_2_ treatment to relative rate constant values-k1 for the absorbance-time fitted curves of LcPIP2;4 yeast spheroplasts. Data from six independent experiments and fitted to two-phase exponential decay functions. Lowercase letters indicated significant differences among the data based on one-way ANOVA and Tukey’s multiple range tests (*P* < 0.05).

To determine the role of LcPIPs in the response to water loss and browning and the effect of aquaporin inhibitors HgCl_2_ on the expression, we measured their transcription levels in the litchi pericarps after harvest. Transcriptional analysis revealed that LcPIP2;4 exhibited significantly higher expression levels in litchi pericarps compared to other LcPIP members, with transcription levels 978-fold higher than LcPIP1;4 (Fig. 2A). During postharvest storage, progressive pericarp browning coincided with the downregulation of eight *LcPIP* genes, excluding *LcPIP1;3*, suggesting a potential link between their suppression and browning development. In HgCl₂-treated groups, the downregulation of *LcPIP1;1*, *LcPIP1;2*, *LcPIP1;4*, *LcPIP2;3*, *LcPIP2;4*, and *LcPIP2;5* was significantly more pronounced than in the control group (Fig. S2 and Fig. 2B). Specifically, on Day 2 of storage, *LcPIP1;1*, *LcPIP2;3*, and *LcPIP2;4* were significantly downregulated; on Day 4, *LcPIP1;4* and *LcPIP2;4* showed substantial suppression; and on Day 6, *LcPIP1;2* and *LcPIP2;4* were notably reduced (Fig. S2 and Fig. 2B). *LcPIP2;4* exhibited the most significant downregulation, with expression decreases of 22.94%, 52.49%, and 48.24% on Days 2, 4, and 6, respectively (Fig. 2B), highlighting its pivotal role in HgCl₂-mediated browning regulation.

To assess the impact of HgCl₂ on water transport activity, a yeast spheroplast hypo-osmotic shock assay was performed [26]. Water channel activity was quantified by measuring the reduction in optical absorbance (OD_600_) and calculating the relative rate constant (k₁) from biphasic exponential decay curve fitting. Spheroplasts expressing LcPIP2;4 exhibited a rapid decline in absorbance, decreasing from 1.0 to 0.52 (a 48% reduction) within 1 minute of hypo-osmotic treatment (Fig. 2C). The k₁ value for LcPIP2;4 reached 2.96, significantly higher than that of other LcPIP isoforms (Fig. 2D), confirming its superior water transport capacity. After pre-treatment with 200 μM HgCl₂, the absorbance decline for LcPIP2;4-expressing spheroplasts was reduced to 0.31 (compared to 0.48 in controls), while the k₁ value decreased to 1.80 (compared to 2.96 in controls) (Fig. 2E and F), indicating HgCl₂-mediated inhibition of LcPIP2;4 aquaporin activity. These findings establish LcPIP2;4 as a key regulator in HgCl₂-induced water deficit stress and pericarp browning during postharvest storage.

### LcPIP2;4 positively regulated browning and water loss in litchi fruit and embryonic callus

The *LcPIP2;4* gene was cloned from the cDNA of ‘Feizixiao’ litchi, and sequencing analysis revealed that its full-length CDS is 861 bp, encoding 287 amino acids. Subcellular localization in tobacco (Method S1) demonstrated that LcPIP2;4 is located in the cell membrane (Fig. S3A). Transmembrane domain prediction indicated that the protein contains six transmembrane segments (Fig. S3B), confirming its classification as a membrane protein.

**Figure 3:**
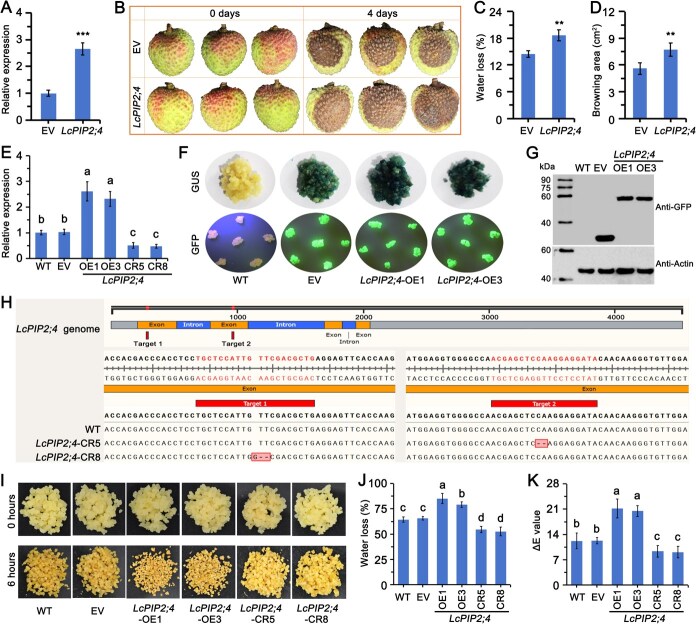
LcPIP2;4 positively regulated browning and water loss in litchi fruit and embryonic callus. (A) Relative expression of LcPIP2;4, (B) phenotype, (C) water loss, and (D) browning area following transient expression of LcPIP2;4 in litchi fruit after 4 days at room temperature. EV means litchi fruits transiently expressing the empty pCAMBIA1301 vector (control group). LcPIP2;4 means litchi fruits transiently overexpressing LcPIP2;4. Data were obtained from three independent experiments, which are shown as means ± SDs. Asterisks (**, and ***) indicate significant differences at *P* < 0.01, and *P* < 0.001, respectively (*t*-test). (E) Relative expression of *LcPIP2;4* in *LcPIP2;4-*overexpressing and CRISPR/Cas9-edited transgenic embryonic callus. (F) GUS staining and GFP fluorescence, and (G) anti-GFP immunoblotting analysis in *LcPIP2;4*-overexpressing transgenic embryonic callus. (H) DNA sequencing analysis showing genomic DNA editing status in CRISPR/Cas9-edited transgenic embryonic callus. (I) Phenotype, (J) water loss, and (K) chromatism value in *LcPIP2;4-*overexpressing and CRISPR/Cas9-edited transgenic embryonic callus. Data obtained from three independent experiments, which are shown as means ± SDs. Lowercase letters indicated significant differences among the data based on one-way ANOVA and Tukey’s multiple range tests (*P* < 0.05).

To investigate the role of LcPIP2;4 in water loss and browning of ‘Feizixiao’ litchi fruit, we transiently expressed the gene in litchi fruit via *Agrobacterium* infiltration. The expression level of LcPIP2;4 in the transiently transformed litchi fruit was 2.66 times higher than that in the empty vector (EV) control (Fig. 3A). After 4 days of postharvest storage, the browning in the *LcPIP2;4* transient expression group was significantly more severe than in EV (Fig. 3B). The water loss rate and browning area were 1.29 and 1.38 times greater, respectively, than in the EV (Fig. 3C and D), indicating that LcPIP2;4 promotes water loss and browning in litchi fruit.

Transgenic embryonic callus generated via Agrobacterium-mediated transformation were employed for functional validation of LcPIP2;4. We successfully generated *LcPIP2;4* overexpression (OE) and CRISPR/Cas9 knockout (CR) ‘Feizixiao’ litchi embryogenic callus (Fig. 3E–H). The expression of *LcPIP2;4* in the *LcPIP2;4*-OE callus was significantly higher than in wild-type (WT) and EV controls, whereas the expression in the *LcPIP2;4*-CR was notably reduced (Fig. 3E). GUS histochemical staining (Fig. 3F), GFP fluorescence detection (Fig. 3F), and anti-GFP Western blotting (Fig. 3G) confirmed successful expression of the LcPIP2;4-GFP fusion protein. CRISPR/Cas9 editing results revealed that *LcPIP2;4*-CR5 had a 2-base pair deletion at target site 2, whereas *LcPIP2;4*-CR8 had a 1-base insertion and a 2-base deletion at target site 1 (Fig. 3H). After 6 hours, the degree of browning in the *LcPIP2;4*-OE callus was significantly greater than in WT and EV controls, while the *LcPIP2;4*-CR exhibited less browning (Fig. 3I). Water loss rate analysis revealed that *LcPIP2;4*-OE1 and *LcPIP2;4*-OE3 callus had significantly higher water loss rates than WT and EV controls, with rates 1.33 and 1.21 times higher than EV, respectively. In contrast, the water loss rates in *LcPIP2;4*-CR5 and *LcPIP2;4*-CR8 callus were significantly lower than in the WT and EV controls, being only 83.52% and 80.45% of the EV, respectively (Fig. 3J). Analysis of browning (measured by chromatism value) revealed that *LcPIP2;4*-OE1 and *LcPIP2;4*-OE3 callus exhibited more pronounced color changes, with their chromatism values 1.72 and 1.66 times higher than the EV control, respectively. In contrast, the chromatism (Δ*E*) value of *LcPIP2;4*-CR5 and *LcPIP2;4*-CR8 callus were significantly lower than those of WT and EV, being only 76.07% and 74.15% of the EV, respectively (Fig. 3K). These results collectively suggest that LcPIP2;4 promotes water loss and browning in litchi callus.

### LcMYB306 regulates LcPIP2;4 transcription by binding to the promoter

**Figure 4:**
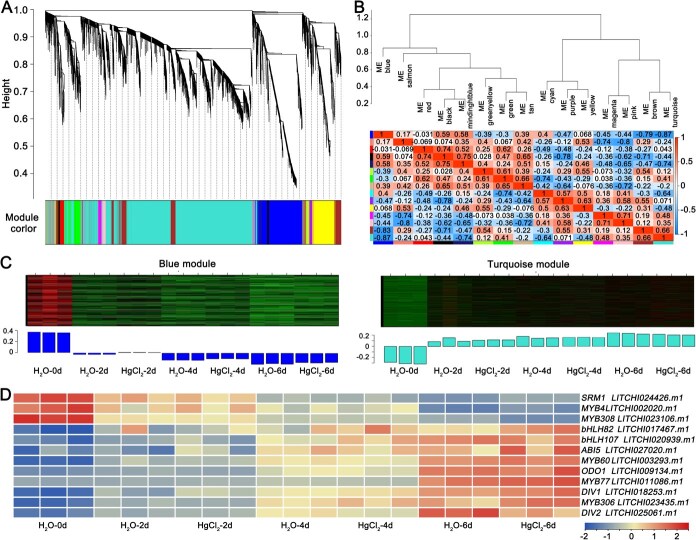
WGCNA analysis, combined with LcPIP2;4 promoter element analysis, identified transcription factors potentially associated with the expression of LcPIP2;4 during the water loss-induced browning of litchi pericarps. (A) Dendrogram of hierarchical clustering for the modules. (B) Heatmap of correlations between modules; (C) Gene expression patterns of the module containing LcPIP2;4 and its significantly correlated module. (D) Heatmap of potential transcription factors identified through WGCNA and LcPIP2;4 promoter element analysis. Samples include the control group (0, 2, 4, 6 days post-harvest litchi pericarps) and the HgCl₂-treated group (2, 4, 6 days post-treatment litchi pericarps) and data from RNA sequencing.

To identify the regulatory factors of LcPIP2;4, transcriptome sequencing was conducted on 21 samples, generating 144.23 Gb of clean data. Following the construction of a gene co-expression network using WGCNA, a clustering dendrogram was created based on gene expression correlations, resulting in the division of the network into 15 distinct modules (Fig. 4A). The analysis indicated that the gene expression pattern of LcPIP2;4 is associated with the blue module. A module correlation heatmap revealed a significant negative correlation between the blue and turquoise modules (*r* = −0.87) (Fig. 4B and C). In these modules, 50 transcription factors were identified, including those from families such as AP2, bHLH, bZIP, C3H, and MYB (Table S3). Furthermore cis-element analysis of the LcPIP2;4 promoter revealed the presence of binding sites for bHLH (G-box), bZIP (ABRE), and MYB (Table S4). Combined analysis ultimately identified 12 potential regulatory factors (Fig. 4D**,**  Tables S3 and S4). Quantitative Reverse Transcription Polymerase Chain Reaction (qRT-PCR) validation showed that the expression patterns of 10 transcription factors (*LcSRM1*, *LcMYB4*, *LcMYB308*, *LcbHLH82*, *LcbHLH108*, *LcMYB60*, *LcODO1*, *LcMYB77*, *LcMYB306*, and *LcDIV2*) were consistent with the results of transcriptome (Fig. S4**,**  Fig. 4D).

We performed a yeast one-hybrid (Y1H) assay (Method S2) to investigate the interaction between 10 potential transcription factors and the *LcPIP2;4* promoter. The results showed that only the co-transformed strain pADT7-LcMYB306 + pHIS2-proLcPIP2;4 was able to grow on SD/-Trp-Leu-His medium supplemented with 50 mM 3-amino-1,2,4-triazole (3AT), while the other nine co-expressed strains (pGADT7-LcSRM1 + pHIS2-proLcPIP2;4, pGADT7-LcMYB4 + pHIS2-proLcPIP2;4, pGADT7-LcMYB308 + pHIS2-proLcPIP2;4, pGADT7-LcbHLH82 + pHIS2-proLcPIP2;4, pGADT7-LcbHLH108 + pHIS2-proLcPIP2;4, pGADT7-LcMYB60 + pHIS2-proLcPIP2;4, pGADT7-LcODO1 + pHIS2-proLcPIP2;4, pGADT7-LcMYB77 + pHIS2-proLcPIP2;4, and pGADT7-LcDIV2 + pHIS2-proLcPIP2;4) failed to grow (Fig. S5). This indicates that LcMYB306 interacts with the LcPIP2;4 promoter.

To assess the binding specificity, we used the Y1H method to examine the interaction between LcMYB306 and the promoters of eight other LcPIP promoters (*LcPIP1;1*, *LcPIP1;2*, *LcPIP1;3*, *LcPIP1;4*, *LcPIP2;1*, *LcPIP2;2*, *LcPIP2;3,* and *LcPIP2;5*). The results revealed that all co-expressed strains failed to grow on SD/-Trp-Leu-His medium supplemented with 3AT (Fig. S6), confirming that LcMYB306 does not interact with the promoters of the other eight *LcPIP* promoters.

**Figure 5:**
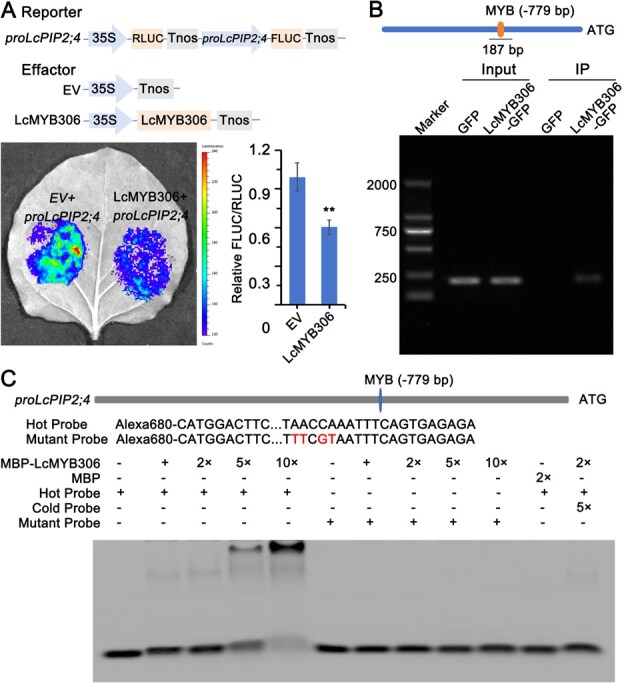
LcMYB306 can bind to *LcPIP2;4* prompter both *in vitro* and *in vivo*. (A) Dual-luciferase reporter assay confirmed the binding of LcMYB306 to the *LcPIP2;4* promoter in tobacco leaves. The biofluorescence produced by firefly luciferase and D-luciferase potassium salt was detected by a plant live imaging system. The error bars represent the standard deviation of six replicates. Asterisks (**) indicate significant differences at *P* < 0.01 (*t*-test). (B) ChIP-PCR assay demonstrated LcMYB306 binding to the LcPIP2;4 promoter in litchi embryonic callus. Input, DNA fragments from chromatin extracts before immunoprecipitation; IP, DNA fragments from chromatin extracts precipitated using the specific antibody α-GFP; GFP, ‘Feizixiao’ litchi embryonic callus expressing GFP; LcMYB306-GFP, ‘Feizixiao’ litchi embryonic callus expressing LcMYB306-GFP. (C) EMSA assay showing the specific binding of LcMYB306 to LcPIP2;4 promoter *in vitro*. Alexa 680-labeled *LcPIP2;4* promoter fragment with MYB motif was used as hot probe, containing mutated bases at the MYB motif as mutant probe, and unlabeled as cold probe. MBP, MBP protein was expressed in *Escherichia coli* BL21 (DE3)；MBP-LcMYB306，MBP-LcMYB306 fusion protein was expressed in *Escherichia coli* BL21 (DE3).

To further confirm LcMYB306 directly regulate to the expression of LcPIP2;4, a dual-luciferase reporter assay was performed using tobacco leaves. Compared to the EV + *proLcPIP2;4*, the relative luciferase activity (FLUC/RLUC) in the co-transfected LcMYB306 + *proLcPIP2;4* group was significantly reduced (Fig. 5A), confirming that LcMYB306 suppresses *LcPIP2;4* transcription.

Chromatin immunoprecipitation (ChIP)-Polymerase Chain Reaction (PCR) and electrophoretic mobility shift assay (EMSA) assays were performed to validate specific binding targets of protein LcMYB306 and DNA element LcPIP2;4 promoter. In ‘Feizixiao’ embryogenic callus expressing the LcMYB306-GFP fusion protein, ChIP-PCR was used to examine the binding affinity of LcMYB306 to the promoter of *LcPIP2;4*, which was divided into 11 distinct regions. Only the *proPIP2;4* (−728 to −914 bp) fragment was enriched following immunoprecipitation (Fig. 5B and Fig. S7), indicating that this region serves as the *in vivo* binding site for LcMYB306. Sequence analysis revealed the presence of the MYB core binding motif (TAACCA) within this region (Table S4). EMSA experiments further confirmed the binding specificity, as the concentration of MBP-LcMYB306 protein increased, the band shift intensified when binding to the Hot Probe. In contrast, no shift was observed with the mutated probe (Mutant Probe) (Fig. 5C), confirming that LcMYB306 regulates *LcPIP2;4* through the MYB element at −779 bp.

In summary, LcMYB306 suppresses LcPIP2;4 transcriptional activity by specifically targeting and binding to the LcPIP2;4 promoter region.

### LcMYB306 negatively regulates water loss and browning in litchi by inhibiting LcPIP2;4 expression

**Figure 6:**
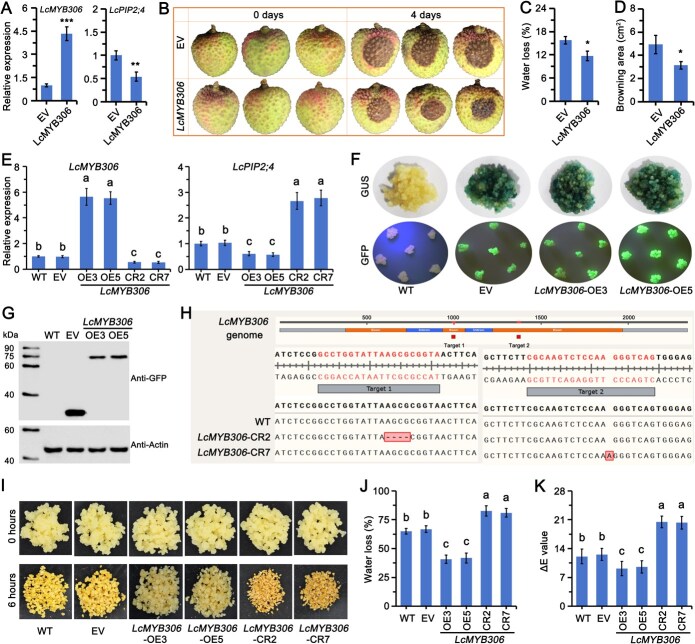
LcMYB306 negatively regulated *LcPIP2;4* expression and inhibiting browning and water loss in litchi fruit and embryonic callus. (A) relative expression of *LcMYB306* and *LcPIP2;4*, (B) Phenotype, (C) water loss, and (D) browning area following transient expression of *LcMYB306* in litchi fruit after 4 days at room temperature. EV means litchi fruits transiently expressing the empty pCAMBIA1301 vector (control group). LcPIP306 means litchi fruits transiently overexpressing *LcMYBL306*. Data were obtained from three independent experiments, which are shown as means ± SDs. Asterisks (^**^, and ^***^) indicate significant differences at *P* < 0.01, and *P* < 0.001, respectively (*t*-test). (E) Relative expression of *LcMYB306* and *LcPIP2;4* in *LcMYB306-*overexpressing and CRISPR/Cas9-edited transgenic embryonic callus. (F) GUS staining and GFP fluorescence, and (G) anti-GFP immunoblotting analysis in *LcMYB306*-overexpressing transgenic embryonic callus. (H) DNA sequencing analysis showing genomic DNA editing status in CRISPR/Cas9-edited transgenic embryonic callus. (I) Phenotype, (J) water loss, and (K) chromatism value in *LcMYB306-*overexpressing and CRISPR/Cas9-edited transgenic embryonic callus. Data obtained from three independent experiments, which are shown as means ± SDs. Lowercase letters indicated significant differences among the data based on one-way ANOVA and Tukey’s multiple range tests (*P* < 0.05).

To investigate the regulatory role of LcMYB306 in *LcPIP2;4* transcription, water metabolism, and browning in litchi fruit, we transiently overexpressed *LcMYB306* using the Agrobacterium infiltration method. The results demonstrated that the transcription level of *LcMYB306* overexpressing group was 4.33 times higher than in the EV control group, while *LcPIP2;4* expression was reduced to 54% of that in the EV group (Fig. 6A), confirming that LcMYB306 suppresses *LcPIP2;4* transcription. After 4 days of post-harvest storage, the overexpression group exhibited significantly less browning compared to the control group (Fig. 6B). The water loss rate and browning area were 74.16% and 63.83% of those in the EV group, respectively (Fig. 6C and D), indicating that LcMYB306 inhibits water loss and browning in litchi fruit.

To validate the functional role of LcMYB306, we generated *LcMYB306*-OE and *LcMYB306*-CR callus through Agrobacterium-mediated genetic transformation assessing its regulatory impact on target gene expression and phenotypic outcomes. In the *LcMYB306*-OE callus *LcMYB306* expression was significantly higher than in the WT and EV groups, while *LcPIP2;4* expression was significantly lower. In contrast, *LcMYB306*-CR callus exhibited the opposite trendency (Fig. 6E), further confirming that LcMYB306 negatively regulates *LcPIP2;4*. GUS staining, GFP fluorescence, and anti-GFP western blot analysis confirmed successful expression of LcMYB306-GFP (Fig. 6F and G). Site editing results revealed a 4-base deletion at target site 1 in *LcMYB306*-CR2, and a 1-base insertion at target site 2 in *LcMYB306*-CR7 (Fig. 6H). After 6 hours, the *LcMYB306*-OE callus showed significantly less browning compared to WT and EV callus whereas the *LcMYB306*-CR exhibited more severe browning (Fig. 6I). Water loss analysis revealed that the water loss rate of *LcMYB306*-OE3 and *LcMYB306*-OE5 callus was significantly lower than that of the WT and EV groups, at 61.80% and 60.67% of the EV group, respectively. In contrast, the water loss rate in *LcMYB306*-CR2 and *LcMYB306*-CR7 callus was significantly higher than in the WT and EV groups, at 1.24 and 1.21 times the rate of the EV group (Fig. 6J). Browning analysis, measured by chromatism, showed minimal color change in *LcMYB306*-OE3 and *LcMYB306*-OE5 callus with chromatism values significantly lower than those of the WT and EV controls, at 72.58% and 75.86% of the EV group, respectively. Conversely, the chromatism values in *LcMYB306*-CR2 and *LcMYB306*-CR7 callus were significantly higher than those in the WT and EV groups, at 1.63 and 1.61 times the EV group values (Fig. 6K). These results collectively suggest that LcMYB306 inhibits water loss and browning in litchi callus by negatively regulating *LcPIP2;4* transcription.

## Discussion

Pericarp browning is a serious postharvest issue in litchi, substantially reducing its commercial value and shelf life. The primary factor inducing browning is postharvest water loss from the pericarp, with the water loss rate positively correlated with the degree of browning [5]. Inhibition of water loss effectively delays peel browning [11, 17]. Water loss from the pericarp results from the transport of water from intracellular spaces to the extracellular matrix, followed by transpiration into the surrounding environment [25]. This cellular-level water transport is primarily regulated by aquaporins (AQPs) [29]. The water channel protein inhibitor HgCl₂ effectively suppresses AQP activity [26–28]. After HgCl₂ treatment, both the water loss rate and browning of litchi pericarp are partially inhibited (Fig. 1), indicating that AQP-mediated water transport influences litchi water loss and browning.

Plasma membrane intrinsic proteins are key aquaporins regulating water transport between the cytoplasm and extracellular matrix [20, 30]. Downregulation of PIP expression in the pericarp may play a crucial role in delaying water loss, as reported in grapefruit, navel orange [31], and yellow-brown sand pears [24], where reduced PIP expression during storage is associated with enhanced water retention and delayed water loss. Moreover, decreased PIP expression has been observed in postharvest sweet orange fruit, and heterologous expression of CsPIP2;4 in kumquat fruit delays water loss [25]. However, direct evidence linking PIP regulation to water loss remains insufficient. In this study, we demonstrate that *LcPIP2;4*, a member of the PIP2 subfamily, is the most abundantly expressed *PIP* gene in litchi pericarp, with its expression significantly downregulated during postharvest storage (Fig. 2A and B). Functional assays reveal that LcPIP2;4 exhibits the strongest water transport activity (Fig. 2C and D). After HgCl₂ treatment, both the expression of LcPIP2;4 and its water transport capacity were suppressed (Fig. 2), suggesting that *LcPIP2;4* as a critical gene regulating HgCl₂-induced dehydration browning in litchi pericarp. By transiently overexpressing *LcPIP2;4* in ‘Feizixiao’ litchi fruit and using transgenic callus tissue, we confirm that LcPIP2;4 positively regulates water loss and browning in litchi (Fig. 3), which provides direct evidence for the role of PIP proteins in regulating pericarp water loss.

This study demonstrates that LcMYB306, a member of the R2R3-MYB transcription factor family, specifically represses LcPIP2;4 transcription through direct interaction with its promoter. The Y1H assay screened 10 candidate TFs, revealing that only LcMYB306 binds the LcPIP2;4 promoter under high-stringency conditions (Fig. S5). Notably, no binding affinity was detected between LcMYB306 and eight other LcPIP promoters (Fig. S6), highlighting its target specificity. This aligns with studies showing that R2R3-MYBs discriminate between similar promoters based on cis-element variations, such as motif composition and spatial arrangement [25, 32]. Functional validation via dual-luciferase assays confirmed LcMYB306-mediated repression of LcPIP2;4 promoter activity (Fig. 5A), while ChIP-PCR localized the binding site to the −728 to −914 bp region of the promoter (Fig. 5B and Fig. S7). Sequence analysis identified a critical TAACAA motif within this region, and EMSA confirmed its necessity for LcMYB306 binding (Fig. 5C and Table S4). Mutating this motif abolished interaction, consistent with the dual regulatory roles of R2R3-MYBs, where promoter context dictates transcriptional activation or repression [25, 32, 33].

The specificity of LcMYB306 for LcPIP2;4 may reflect evolutionary diversification of aquaporin regulatory mechanisms. For instance, analogous R2R3-MYBs like CsMYB96 in citrus repress *CsPIP* genes while activating wax biosynthesis genes (CsCER1), balancing water retention under stress [25]. Similarly, AtMYB60 and VvMYB60 regulate stomatal closure by targeting distinct cis-elements [34, 35]. The absence of TAACAA motifs in other LcPIP promoters likely explains their insensitivity to LcMYB306, mirroring the ‘motif-divergent’ regulatory strategies observed in plant MYB networks [36].

Mechanistically, repression by LcMYB306 may involve co-repressor recruitment via conserved domains or steric hindrance of transcriptional activators [37]. This aligns with studies showing that R2R3-MYB repressors often recruit TPL/TPL-like complexes to suppress target genes [37]. Furthermore, the observed reduced LcPIP2;4 transcripts in transgenic callus (Fig. 6B) underscores the biological relevance of this interaction, potentially mitigating water loss under stress—a strategy paralleled in MdMYB94 (apple) and CsMYB96 (citrus), which enhance drought tolerance via cuticular wax regulation [25, 38, 39]. The isoform-specific repression of LcPIP2;4 by LcMYB306 likely prevents functional redundancy among aquaporins, enabling precise modulation of water transport. Similar regulatory specificity is observed in *Hevea brasiliensis* AP2/ERF TFs, where distinct isoforms regulate latex biosynthesis versus stress responses [40]. This selective repression may optimize cellular water balance under fluctuating environmental conditions, a critical adaptation for litchi growth and stress resilience.

## Conclusion

This study shows that LcMYB306 delays water loss and browning in litchi by repressing the expression of LcPIP2;4, providing new insights into the regulatory mechanisms of post-harvest fruit water loss and browning. These findings have significant implications for maintaining the quality of postharvest fruits.

## Materials and methods

### Plant materials and treatments

Uniformly mature ‘Feizixiao’ fruit, with consistent size, color, and no mechanical damage, were obtained from a local litchi grove in Danzhou, Hainan Province, China. The fruit were transported to the laboratory on the same day of harvest. They were soaked in a 0.05% sodium hypochlorite solution for 30 seconds, washed twice with distilled water, and air-dried prior to use in subsequent experiments.

Fruits were immersed in either distilled water (control group) or a 0.2-mM HgCl_2_ solution (treatment group) and subjected to vacuum infiltration for 30 minutes in a desiccator connected to a vacuum pump. After the infiltration solution was removed, the vacuum was maintained for an additional 10 minutes. The fruits were then removed, air-dried naturally, and placed into perforated fresh-keeping boxes (240 fruits per treatment, divided into three replicates of 80 fruits each). The boxes were incubated at 25°C and 80% relative humidity.

On Days 0, 2, 4, and 6, 15 fruits (three replicates, five fruits per replicate) were sampled. The pericarp was peeled, immediately frozen in liquid nitrogen, and stored at −80°C for transcriptomic and qRT-PCR analysis. Another 15 fruits (three replicates, five fruits per replicate) were used to measure pericarp water loss rate. Additionally, 90 fruits (3 replicates, 30 fruits per replicate) were fixed for continuous color difference analysis.

Embryogenic callus of *Litchi chinensis* cv. ‘Feizixiao’, subcultured for ten generations, were inoculated onto M3 medium (containing 4.43 g MS basal salts, 1 mg 2,4-D, 30 g sucrose, and 7 g agar per liter) and cultured in the dark at 25°C for 20 days for subsequent genetic transformation of *LcPIP2;4* and *LcMYB306*.

*Nicotiana benthamiana* plants were grown under a 16-hour light/8-hour dark photoperiod at 25°C ± 2°C with 60%–70% relative humidity for three weeks and used for dual-luciferase reporter assays.

### Tristimulus color measurements, browning index, and water loss

Color changes (L*, a*, b* values) in litchi fruits and embryogenic callus tissue were measured using a colorimeter (3nh CR8, Shenzhen, China). Measurements were taken on Days 0, 2, 4, and 6, with four points selected from the central region of each fruit. A total of 90 fruits were measured per treatment. The chromatism (ΔE^*^) values was calculated using the following formula: ΔE^*^ = [(L_t_^*^ `− *L*_0_^*^)^2^ + (a_t_^*^ − *a*_0_^*^)^2^ + (b_t_^*^ − *b*_0_^*^)^2^]^1/₂.^ For the embryogenic callus 1.0 g was evenly spread on a culture dish and placed in an environment at 25°C and 80% humidity. Measurements were taken at 0 and 6 hours, with 10 random points measured per culture dish. A total of 6 dishes were measured per treatment. The color difference was calculated using the same formula as for the fruits.

The browning degree of the litchi fruits was assessed using the method described by Shen *et al*. [41], with evaluations performed on Days 0, 2, 4, and 6. The browning degree scale is as follows: 0 (no browning), 1 (browning area ≤ 1/20), 2 (browning area ≤ 1/4), 3 (browning area > 1/4 and ≤ 1/3), 4 (browning area > 1/3 and ≤ 1/2), 5 (browning area > 1/2 and ≤ 3/4), and 6 (browning area > 3/4). The browning index was calculated using the formula: Browning index = Σ (browning grade × number of fruits for that grade) / (6 × total number of fruits).

On Day 0, the pericarp of the litchi fruits was removed (three replicates, five fruits per replicate), and the fresh weight (*m*_1_) was measured. The samples were then dried to a constant weight and weighed again (*m*_0_). On Days 2, 4, and 6, the pericarp was collected using the same method, and the fresh weight (*m_t_*) was measured. The water loss rate was calculated using the following formula: Water loss (%) = (*m_t_* − *m*_0_) / (*m*_1_ − *m*_0_) × 100.

For the embryogenic callus 1.0 g (fresh weight *m*_1_) was spread evenly on a culture dish and placed in an environment at 25°C and 80% humidity for 6 hours. Afterward, the weight (m_2)_ was measured. Take another 1.0 g embryogenic callus and then dried to a constant weight (m_0_). A total of 6 measurements per treatment were taken. The water loss rate was calculated using the formula: Water loss (%) = (*m*_2_ − *m*_0_) / (*m*_1_ − *m*_0_) × 100.

### RNA extraction and real-time fluorescence quantitative analysis

Total RNA was extracted using the RNA extraction kit (Beijing Huayueyang Biotechnology Co., Ltd., Beijing, China). The RNA concentration (A260/A280) and purity were assessed using a spectrophotometer (NanoDrop One, Thermo Fisher Scientific, Waltham, Massachusetts, USA), and the integrity was verified by 1.0% agarose gel electrophoresis (without RNAse) based on the 28S/18S ribosomal RNA band ratio. Quantitative real-time PCR was performed using an ABI 7500 Real-Time Fluorescence PCR System (Applied Biosystems, Carlsbad, CA, USA) combined with SYBR Green dye (Vazyme Biotechnology Co., Ltd., Nanjing, China)**.** The reference gene used was *LcActin*, and specific primers were designed using the NCBI Primer-BLAST tool (https://www.ncbi.nlm.nih.gov/tools/primer-blast/index.cgi?LINK_LOC=BlastHome), with primer sequences, annealing temperatures, and target gene information provided in Table S1. Primer amplification efficiency and relative gene expression (2^−ΔΔCT^) were calculated using LinRegPCR 11.0. Each experiment included three biological and technical replicates to ensure repeatability.

### Water transport assay

The water transport activity of aquaporins was assessed using the yeast heterologous expression system, as described by Nemoto *et al*. [26]. The pYES2-LcPIPs recombinant vector was constructed, and the plasmid was introduced into *Saccharomyces cerevisiae* INVSc1 strain via the LiAc/ssDNA/PEG transformation method. Positive transformants were selected on SD/-Ura-Trp solid medium. A single colony was inoculated into SC liquid medium containing 2% sucrose and incubated at 30°C with shaking for 24 hours to activate the strain. The pre-culture was then transferred to SC medium containing 2% galactose and 1% sucrose (OD_600_ = 0.4) and induced at 30°C for 16 hours to express the protein. The cells were collected, washed with 50 mM sodium phosphate buffer (pH 7.6), resuspended in enzymatic digestion solution (1.2 M sorbitol, 50 mM sodium phosphate buffer, 0.7 mM β-mercaptoethanol, and 10 U Zymolyase-20T), and incubated at 30°C for 1 hour. After centrifugation (5000 rpm, 2 min), the protoplasts were harvested and resuspended in wash buffer (1.2 M sorbitol +50 mM sodium phosphate buffer, pH 7.6). A 100-μl aliquot of the protoplast suspension was added to a cuvette, followed by an equal volume of 50 mM sodium phosphate buffer (pH 7.6). OD_600_ dynamic changes were measured using a XU-6 spectrophotometer (Yipu Instrument Manufacturing Co., Ltd., Shanghai, China). The water transport activity was determined by the relative rate of decrease in OD_600_, with an initial OD_600_ of 1.0 as the baseline. In the inhibitor treatment group, protoplasts were pre-incubated in 200 μM HgCl₂-containing assay buffer for 20 minutes, and activity was measured using the same method. Two-phase exponential decay curves were fitted using GraphPad Prism 8.0 software to calculate the initial rate constant (k₁, rapid permeation phase) and secondary rate constant (k₂, slow equilibrium phase). The results were expressed as the ratio relative to the control group (k₁/Control, k₂/Control), with the experiment independently repeated six times.

### Transient overexpression of LcPIP2;4 and LcMYB306 in litchi fruit

The full-length coding sequences (CDS) of *LcPIP2;4* and *LcMYB306* were cloned into the pCAMBIA1301 vector to generate the recombinant plasmids pCAMBIA1301-LcPIP2;4 and pCAMBIA1301-LcMYB306. Cloning primers are listed in Table S1. The recombinant plasmids were subsequently transformed into *Agrobacterium tumefaciens* GV3101. The inoculation solution was prepared by adding 20 mM MgCl₂, 20 mM 2-(N-morpholino) ethanesulfonic acid, and 100 μM acetosyringone, adjusting the solution to an OD600 of 0.8. Litchi fruit were washed twice with distilled water and allowed to air dry. A small hole was created at the center of each fruit using a 1-ml syringe needle. The fruit was then immersed in the prepared Agrobacterium inoculation solution, placed in a vacuum desiccator, and subjected to vacuum for 20 minutes. After discarding the inoculation solution, the fruits were further subjected to vacuum for an additional 10 minutes. The treated fruits were placed in a storage box, kept in the dark, and incubated at 25°C to 28°C for 4 days. Photographs were taken after 4 days, and the browning area was quantified using ImageJ software. Three biological replicates were performed, with 30 fruits per replicate (90 fruits in total for each treatment group).

### Overexpression and CRISPR/Cas9-mediated knockout of *LcPIP2;4* and *LcMYB306* in ‘Feizixiao’ litchi embryonic callus

The plasmids pCAMBIA1301-LcPIP2;4 and pCAMBIA1301-LcMYB306 were utilized to construct gene overexpressing materials. Gene editing target sites were designed and selected using the GRSPR-GE software (http://skl.scau.edu.cn/). The two target sites of TGCTCCATTGTTCGACGCTG**AGG** (Target 1) and GTATCCTCCTTGGAGCTCGT**TGG** (Target 2) for *LcPIP2;4* to identified. Meantime, two corresponding target sites of TACCGCGCTTAATACCAGGC**CGG** (Target 1) and CGCAAGTCTCCAAGGGTCAG**TGG** (Target 2) for *LcMYB306* to identified. Then, using the designed targets, the plant Cas9/gRNA plasmid construction kit (Catalog No. VK005-14, Viewsolid Biotechnology Co., Ltd., Beijing, China) was employed to generate the gene editing vectors VK005-14-LcPIP2;4-sg1/sg2 and VK005-14-LcMYB306-sg1/sg2.

The transformation procedure was conducted according to a previously established genetic transformation method for litchi embryogenic callus [42]. The vectors were introduced into *Agrobacterium tumefaciens* GV3101. The inoculation solution was prepared using M1 liquid medium (containing 4.43 g MS basal salts, 2 mg 2,4-D and 30 g sucrose) supplemented with 100 μM acetosyringone. The bacterial culture was incubated at 28°C, with OD_600_ adjusted to 0.6 to 0.8, for 2 hours. Embryogenic callus (20 days old, cultured on M3 medium) was sterilized, and excess surface moisture was absorbed using sterile filter paper. The callus was then immersed in the inoculation solution and agitated at 60 rpm for 30 minutes. After the bacterial suspension was discarded, the callus was blotted dry with filter paper. The callus was then placed on M1 solid medium containing 100 μM acetosyringone and incubated in the dark at 25°C for 2 days. The co-cultivated callus was washed five times with M1 liquid medium containing 500 mg/L timentin. Finally, the callus was transferred to M1 solid medium (containing 4.43 g MS basal salts, 2 mg 2,4-D, 30 g sucrose, and 7 g agar per liter) with timentin for three rounds of selection to obtain resistant callus.

### GUS, GFP fluorescence, and protein detection

The GUS staining procedure was performed using the GUS staining kit (G3601, Beijing, China). Transgenic resistant callus (pCAMBIA1301, pCAMBIA1301-LcPIP2;4, pCAMBIA1301-LcMYB306) were used as experimental samples, with wild-type callus serving as the negative control. The callus was immersed in X-Gluc staining solution and incubated overnight at 37°C in the dark. After incubation, the callus was washed with 70% ethanol until the background became colorless, and the staining phenotypes were observed and recorded.

For these callus samples, GFP fluorescence was observed in vivo using a LUYOR-3415RG excitation light source (Shanghai Luyor Co., LTD, Shanghai, China). Anti-GFP immunoblotting was performed to assess the expression of GFP, LcPIP2;4-GFP, and LcMYB306-GFP proteins in the callus. Western blotting was carried out using Beyotime Biotechnology’s Western cell lysis buffer (P0013, Beyotime, Shanghai, China), Anti-GFP Mouse Antibody (HT801, TransGen, Beijing, China), Anti-Mouse IgG(H + L)-HRP (HS201, TransGen, Beijing, China), and Actin (plant-specific) Mouse mAb (AC009, ABclonal, Wuhan, China). The experimental procedure followed previously established protocols [43].

### RNA sequencing and analysis

Litchi pericarp samples stored at −80°C were subjected to RNA sequencing, including both the control group (0, 2, 4, 6 days post-harvest) and the HgCl₂-treated group (2, 4, 6 days post-treatment) by Wuhan MetWare Biotechnology Co., Ltd. Total RNA was quantified using a Qubit 4.0 Fluorometer/MD Microplate Reader and assessed for quality using a Qsep400 Bioanalyzer. PolyA-tailed mRNA was enriched using oligo (dT) magnetic beads, followed by fragmentation, double-stranded cDNA synthesis, and bead-based purification to construct Illumina sequencing libraries. After sequencing on Illumina platforms, clean reads were obtained through quality control processing, including filtration of low-quality reads, evaluation of sequencing error rates, and analysis of GC content distribution.

The clean reads were aligned to the reference genome using HISAT2, and gene expression levels were quantified using feature Counts based on FPKM. For weighted gene co-expression network analysis (WGCNA), the expression matrix was preprocessed with the var Filter function (R gene filter package) to remove low-abundance genes (FPKM <1 across all samples) and invariant genes. A scale-free network topology was established using the pick Soft Threshold function (R WGCNA package), with soft thresholding power optimized to achieve scale independence (R^2^ ≥ 0.85).

### Dual-luciferase reporter assay, ChIP analysis, and EMSA

The LcPIP2;4 promoter was cloned into the dual-luciferase reporter vector pGreen II 0800-LUC to generate the reporter vector. The CDS sequence of LcMYB306 was cloned into the pCAMBIA1300 vector to construct the effector vector. Both the reporter and effector vectors were co-transformed into *Agrobacterium tumefaciens* GV3101(pSoup) and used to infect *Nicotiana benthamiana* leaves. After 48–72 hours, D-luciferin solution (ST196, Beyotime, Shanghai, China) was applied to the abaxial surface of the leaves, followed by dark incubation for 3–5 minutes. Imaging was performed using the plant in vivo imaging system (LUMAZONE SOPHIA, Princeton Instruments, USA). The relative activity ratio of FLUC/RLUC was measured using the Dual-Luciferase Reporter Assay Kit (11402ES60, Yeasen Biotechnology, Shanghai, China). The experiment was conducted with six biological replicates.

ChIP analysis was performed on LcMYB306-GFP callus tissues following the protocol outlined in previous studies [44], using the EpiQuik Plant ChIP Kit (P-2014-48, Epigentek, Farmingdale, NY, USA), with GFP-expressing callus tissues serving as the negative control. Anti-GFP antibody was used to enrich target DNA fragments. The callus tissues were vacuum infiltrated with 1% formaldehyde solution for 10 minutes to crosslink DNA-protein complexes, followed by the addition of 0.125 mol·L^−1^ glycine to terminate the reaction. After grinding in liquid nitrogen and centrifugation with lysis buffer (14 000 rpm, 4°C, 45 minutes), chromatin pellets were collected. The chromatin was sonicated to obtain DNA fragments and divided into two groups: the Input group (chromatin supernatant directly used as the template) and the IP group (chromatin fragments incubated with Anti-GFP antibody for 90 minutes, followed by DNA purification using a resin column). PCR was performed with promoter-specific primers (Table S1).

For the EMSA as described by Wang *et al*. [45], the LcPIP2;4 promoter DNA fragment was amplified using Alexa-680 fluorescent-labeled primers and used as a probe. The MBP-LcMYB306 fusion protein was expressed in *Escherichia coli* BL21 (DE3) and purified using Anti-MBP magnetic beads (P2123, Beyotime, Shanghai, China). The purified protein was incubated with 50 ng of labeled probe in binding buffer (GS009-1, Beyotime, Shanghai, China) at 25°C for 20 minutes. The protein-DNA complexes were separated by 0.8% agarose gel electrophoresis and detected using a multifunctional gel imaging system (Azure600, Azure Biosystems, USA) to assess protein-DNA binding specificity.

### Statistical analysis

The data were statistically analyzed using DPS 9.01 (Hangzhou Ruifeng Information Technology Co., LTD, Hangzhou, China) software. The results are presented as the mean ± standard deviation. One-way analysis of variance (ANOVA) was used to assess inter-group differences, and significance was further compared using independent samples two-tailed t-test and Turkey’s multiple range test (*P* < 0.05).

## Supplementary Material

Web_Material_uhaf322

## Data Availability

The raw RNA-seq data generated from this study have been submitted to National Genomics Data Center and the accession number is PRJCA044708. Sequence data can be found in the SapBase (http://www.sapindaceae.com/index.html) under the following accession numbers: *LcPIP1;1* (*LITCHI018243.m1*), *LcPIP1;2* (*LITCHI022094.m1*), *LcPIP1;3* (*LITCHI027537.m1*), *LcPIP1;4* (*LITCHI025728.m1*), *LcPIP2;1* (*LITCHI027285.m1*), *LcPIP2;2* (*LITCHI002040.m1*), *LcPIP2;3* (*LITCHI018583.m1*), *LcPIP2;4* (*LITCHI024675.m1*), *LcPIP2;5* (*LITCHI027284.m1*), *LcSRM1* (*LITCHI024426.m1*), *LcMYB4* (*LITCHI002020.m1*), LcMYB308 (*LITCHI023106.m1*), *LcbHLH82* (*LITCHI017467.m1*), *LcbHLH108* (*LITCHI020939.m1*), *LcMYB60* (*LITCHI003293.m1*), *LcODO1* (*LITCHI009134.m1*), *LcMYB77* (*LITCHI011086.m1*), LcMYB306 (*LITCHI023435.m1*), and *LcDIV2* (*LITCHI025061.m1*).
