# Supplementary material for: LcMYB306 regulates litchi fruit water loss and browning by inhibiting the expression of LcPIP2;4
Source: Hortic Res. 2025 Nov 21;13(3):uhaf322. doi: 10.1093/hr/uhaf322 (PMC12966010; doi:10.1093/hr/uhaf322)
Supplement: Web_Material_uhaf322 [file web_material_uhaf322.docx]

**Title: LcMYB306 regulates litchi fruit water loss and browning by inhibiting the expression of *LcPIP2;4***

Xiaoxu Li ^a^, Fang Li ^a^, Guo Wang ^a^, Shujun Wang ^a^, Xueren Cao ^a^, Ya Wu ^a^, Huanling Li ^a, *^, Jiabao Wang ^a, *^

^a^ National Key Laboratory for Tropical Crop Breeding, Key Laboratory of Integrated Pest Management on Tropical Crops, Ministry of Agriculture and Rural Affairs, Hainan Key Laboratory for Monitoring and Control of Tropical Agricultural Pests, Environment and Plant Protection Institute, Chinese Academy of Tropical Agricultural Sciences, Haikou 571101, China;

^*^ Correspondence authors: lihuanling115@catas.cn (H. Li); wangjiabao@catas.cn (J. Wang)

Other authors: lixiaoxu@catas.cn (X. Li); lifang200709@catas.cn (F. Li); wangguo@catas.cn (G. Wang); Wshujun86@catas.cn (S. Wang); caoxueren1984@catas.cn (X. Cao); wuya@catas.cn (Y. Wu)

**Short running head:** LcMYB306-LcPIP2;4 regulate litchi browning

**Method S1. Subcellular localization analysis**

The recombinant plasmid pCAMBIA1300-LcPIP2;4-GFP was constructed and introduced into *Agrobacterium tumefaciens* GV3101. The infiltration solution was prepared with 20 mM MgCl₂, 20 mM MES, and 100 μM acetosyringone, and the infection suspension was adjusted to an optical density (OD) of 0.5 at 600 nm. After incubating for 2–3 hours at 28°C in the dark, the solution was infiltrated into the leaves of 3 weeks *Nicotiana benthamiana* leaves. Green fluorescence signals were observed 48 hours post-infiltration using a laser confocal microscope (FV1000, Olympus, Japan).

**Method S2. Yeast one-hybrid assay**

The bait vector pHIS2-proLcPIP2;4 and prey vectors pGADT7-TFs (including pGADT7-LcSRM1, pGADT7-LcMYB4, pGADT7-LcMYB308, pGADT7-LcbHLH82, pGADT7-LcbHLH108, pGADT7-LcMYB60, pGADT7-LcODO1, pGADT7-LcMYB77, pGADT7-LcMYB306, and pGADT7-LcDIV2) were constructed. The pHIS2-LcPIP2;4pro plasmid was transformed into the Y187 yeast strain and plated on SD/-Trp medium, followed by incubation at 30°C for 3 days. Single colonies were randomly selected, serially diluted, and inoculated onto SD/-Trp-His medium containing 0-75 mM 3-amino-1,2,4-triazole (3AT) to determine the optimal 3AT concentration for inhibiting background growth, with incubation at 30°C for 3 days. The bait and prey vectors were co-transformed into the Y187 yeast strain and plated on SD/-Leu-Trp agar plates, followed by incubation for 3 days. Yeast cells were then collected, resuspended in deionized water to an optical density (OD) of 0.5 at 600 nm, and spotted onto SD/-Leu-Trp-His medium containing 3AT. Transcription factors interacting with the LcPIP2;4 promoter were identified based on colony growth.


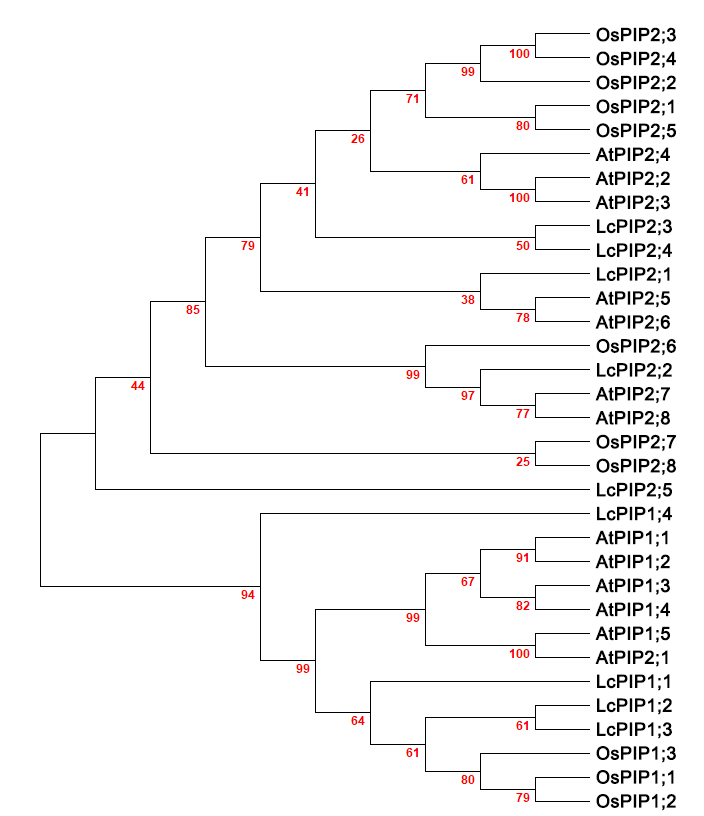


**Figure S1.** A phylogenetic tree of AtPIP, OsPIP and LcPIP protein sequences constructed by the neighbor-joining method.


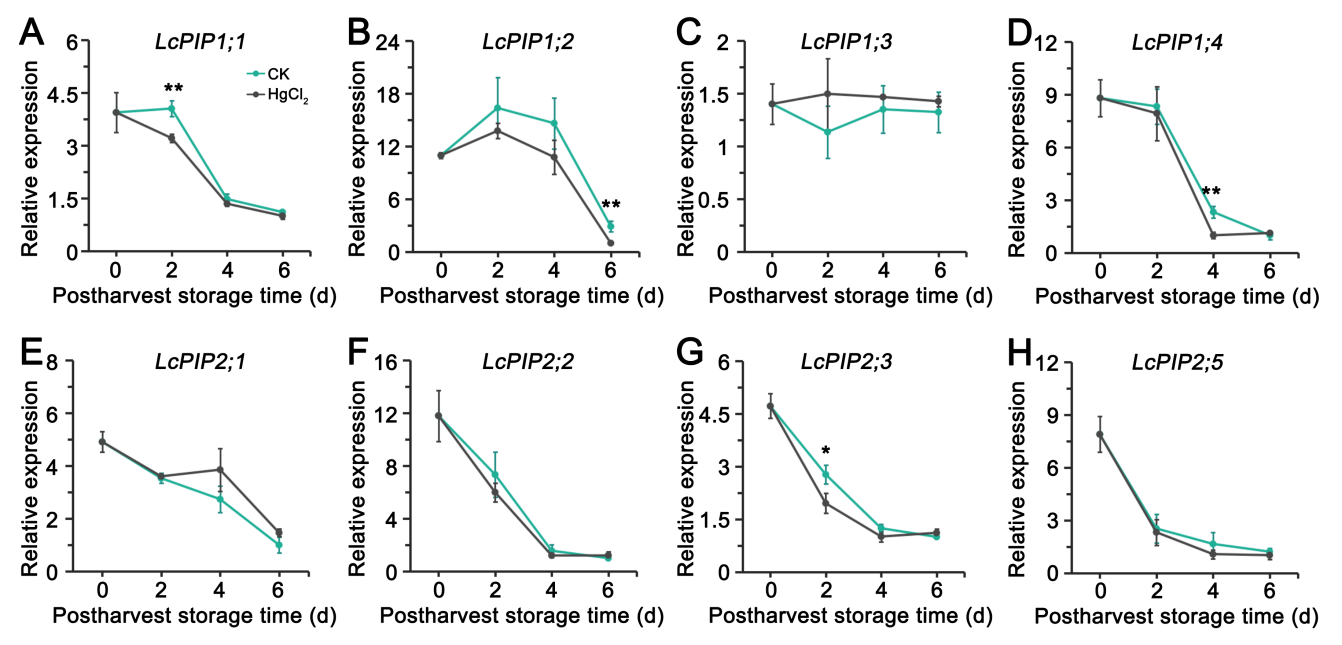


**Figure S2.** The relative expression of eight *LcPIPs* in litchi pericarps was assessed by qRT-PCR during postharvest storage. Asterisks (* and **) indicate significant differences at *P* < 0.05 and *P* < 0.01, respectively (t-test).


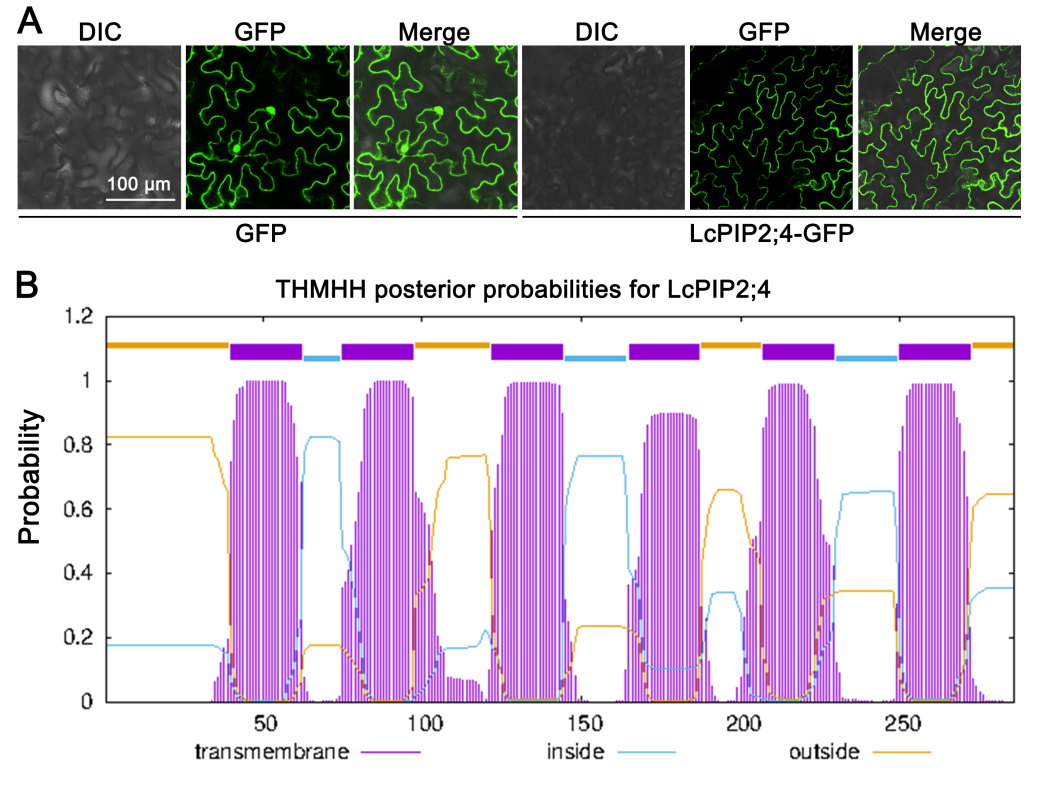


**Figure S3.** (A) Subcellular localization of LcPIP2;4-GFP in *Nicotiana benthamiana* for 48 h by laser scanning confocal microscopy (LSCM) and (B) transmembrane domain of LcPIP2;4 predicted by TMHMM 2.0.


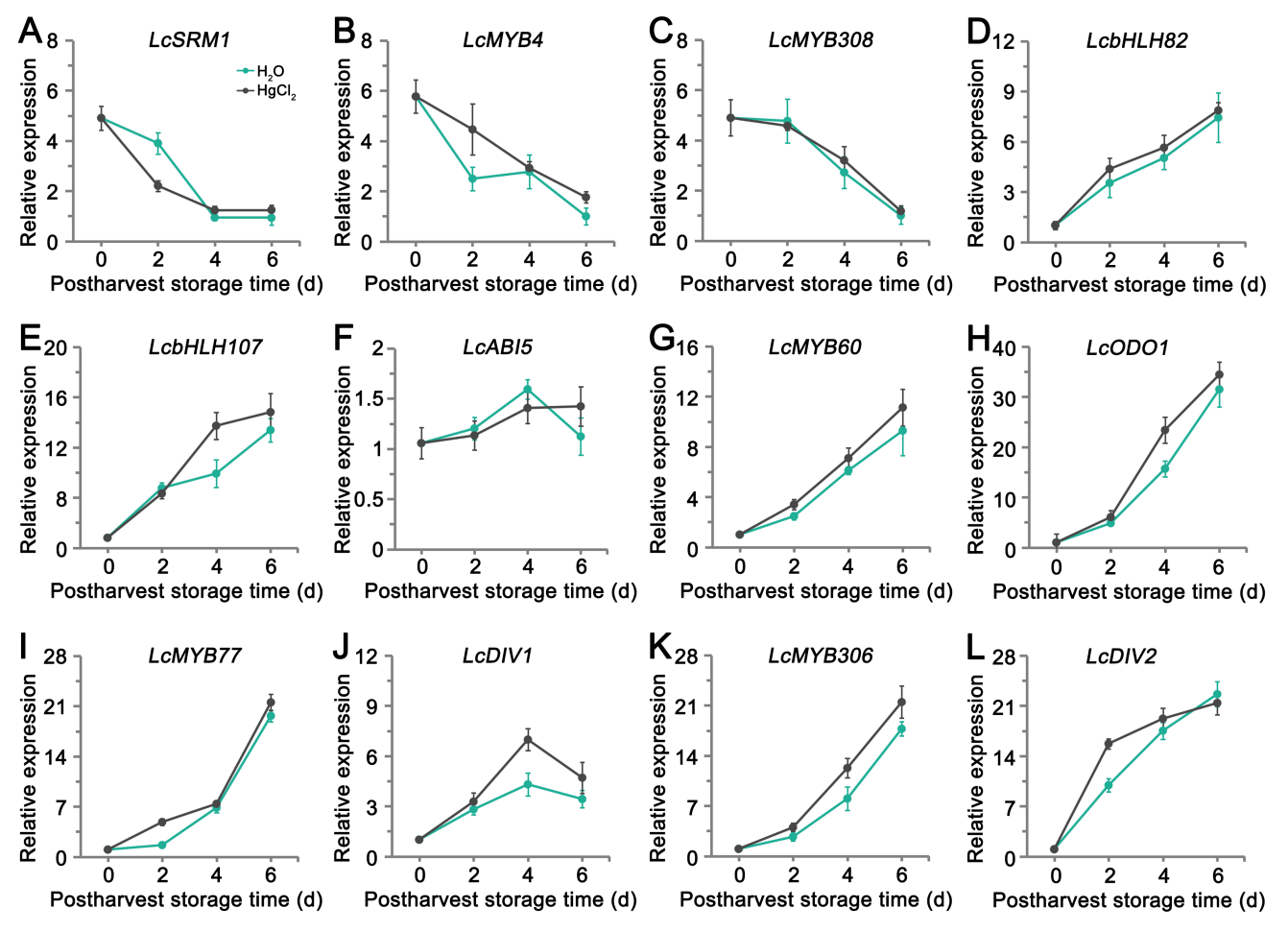


**Figure S4.** The relative expression of 12 potential transcription factors in litchi pericarps was assessed by qRT-PCR during postharvest storage.


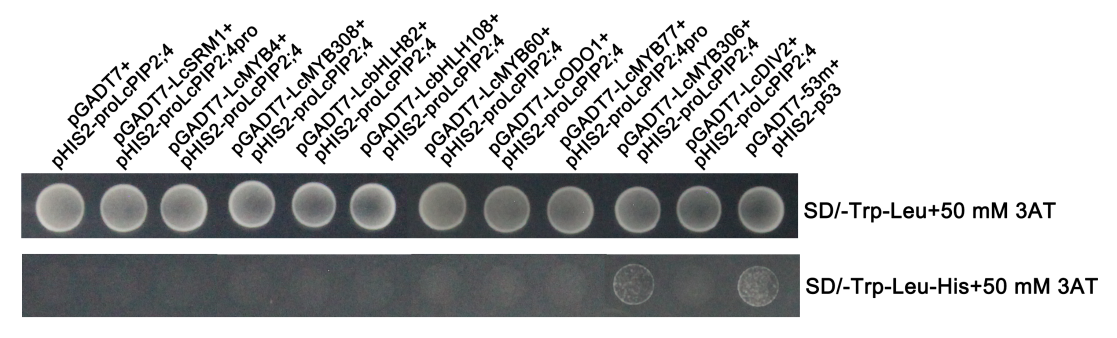


**Figure S5.** Yeast one-hybrid assay screening the interaction between LcPIP2;4 promoter and potential regulatory factors. pGADT7+pHIS2-proLcPIP2;4 as negative control and pGADT7-53m+pHIS2-p53 as positive control.


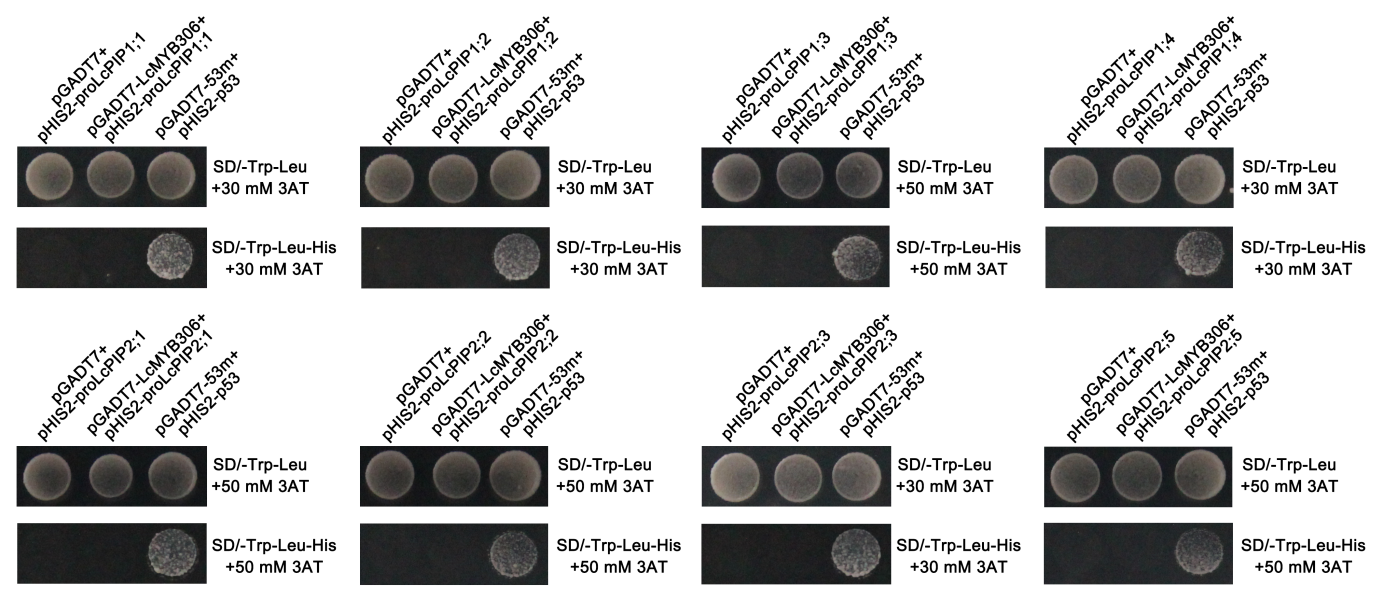


**Figure S6.** Yeast one-hybrid assay examining the interaction between LcMYB306 and the promoters of eight *LcPIP* genes. pGADT7+pHIS2-proLcPIPs as negative control and pGADT7-53m+pHIS2-p53 as positive control.


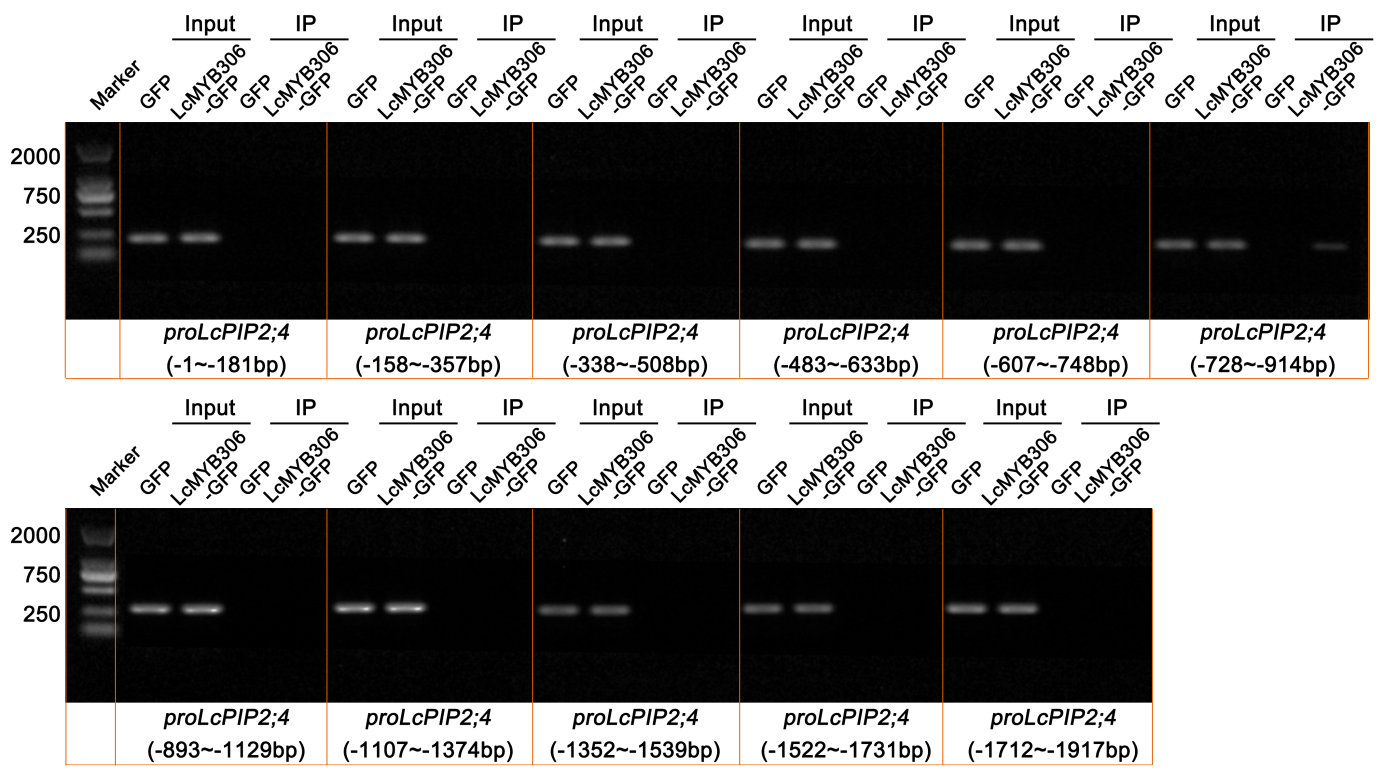


**Figure S7.** ChIP-PCR analysis the binding affinity of LcMYB306 the LcPIP2;4 promoter segments. **ead:** LcMYB306-LcPIP2;4 regulate litchi browning

**Table S1. Primers used in this study**

| Primer name | Primer sequence (5'-3') | Purpose |
| --- | --- | --- |
| Q-LcbHLH82-F | GGACTGCTTCCTACTCCAC | qRT-PCR |
| Q-LcbHLH82-R | GATGATGTATCTTGGGCTGC |  |
| Q-LcbHLH107-F | TGTACGACCGACACTGGCAC |  |
| Q-LcbHLH107-R | GCCTGCACAAGCTCTCCTCT |  |
| Q-LcABI5-F | GGGAGGAAGAGAGGCACTCCA |  |
| Q-LcABI5-R | GTGTAAGCCTGCTTCCTTGC |  |
| Q-LcSMR1-F | TTCCTGGGCGTCATCGTGTT |  |
| Q-LcSMR1-R | GCGATCTTCTCCCACCTCCC |  |
| Q-LcMYB4-F | GGGGAGGTCTCCATGTTGTG |  |
| Q-LcMYB4-R | TTAGGCAGTGAACGCCAGC |  |
| Q-LcMYB60-F | GGGTAACAAATGGGCAGCC |  |
| Q-LcMYB60-R | GTGTCACATGGCCATGCG |  |
| Q-LcODO1-F | CGCCTCCGCTGGACTAACTA |  |
| Q-LcODO1-R | GGACCACCTATTGCCAAGGT |  |
| Q-LcMYB77-F | GGAATGCCCTAGCTCAAGGG |  |
| Q-LcMYB77-R | GCTACCACCTCAAGTTCCTCC |  |
| Q-LcDIV1-F | GATACGCCTGACCGTTGG |  |
| Q-LcDIV1-R | GCCAGGAATTGGAATCAGTCC |  |
| Q-LcMYB308-F | CCACATGGTTACCAATGGCG |  |
| Q-LcMYB308-R | CAAGGTCTTGGCATGTTGCT |  |
| Q-LcMYB306-F | TCCGGCCTGGTATTAAGCGC |  |
| Q-LcMYB306-R | GGCTGCCCATCTGTTACCT |  |
| Q-LcDIV2-F | ACTGAAGTGATCTCCCCTGC |  |
| Q-LcDIV2-R | CCTTGTCATACACAGCCAGAGC |  |
| Q-LcPIP2;4-F | CCCATGTTCCTGTGTTGGCAC |  |
| Q-LcPIP2;4-R | GCGGCTCCAAAACTCCTAGC |  |
| Q-LcMYB306-F | GACCACCTTGCTGTGACAAGA |  |
| Q-LcMYB306-R | CTAAGCAAACCTGTATTGGCAGG |  |
| Q-LcActin-F | TTGGATTCTGGTGATGGTGTG |  |
| Q-Actin-R | CAGCAAGGTCCAACCGAAG |  |
| LcPIP1;1-F(pYES2-LcPIP1;1) | TTGGTACCGAGCTCGGATCCATGAGTGAGATAAAGTCATGGTCTTTCTG | Water transport assay |
| LcPIP1;1-R(pYES2-LcPIP1;1) | GATGGATATCTGCAGAATTCTCAAGCTTTGGACTTGAATGGTTTTGG |  |
| LcPIP1;2-F(pYES2-LcPIP1;2) | TTGGTACCGAGCTCGGATCCATGGAGGGCAAGGAAGAGGAT |  |
| LcPIP1;2-R(pYES2-LcPIP1;2) | GATGGATATCTGCAGAATTCTTAGGCCCTGGTCTTGAATGGAAT |  |
| LcPIP1;3-F(pYES2-LcPIP1;3) | TTGGTACCGAGCTCGGATCCATGGAGGACAAAGAGGAGGATGTT |  |
| LcPIP1;3-R(pYES2-LcPIP1;3) | GATGGATATCTGCAGAATTCTCACTTTGATTTGAAGGGAATGGCTC |  |
| LcPIP1;4-F(pYES2-LcPIP1;4) | TGGTACCGAGCTCGGATCCATGGAGGTTGGAGAAGACGAGAG |  |
| LcPIP1;4-R(pYES2-LcPIP1;4) | GATGGATATCTGCAGAATTCTCAAGCTCTAGACTTGAATGGTTTTGGT |  |
| LcPIP2;1-F(pYES2-LcPIP2;1) | TTGGTACCGAGCTCGGATCCATGGCCAAGGACACGGTG |  |
| LcPIP2;1-R(pYES2-LcPIP2;1) | GATGGATATCTGCAGAATTCTTAGACATGGGACTGGCTCCTG |  |
| LcPIP2;2-F(pYES2-LcPIP2;2) | TTGGTACCGAGCTCGGATCCATGTCGAAGGAAGTGAGTGAAGAAGG |  |
| LcPIP2;2-R(pYES2-LcPIP2;2) | GATGGATATCTGCAGAATTCTCAGTTGGTGGGGTTGCTCC |  |
| LcPIP2;3-F(pYES2-LcPIP2;3) | TTGGTACCGAGCTCGGATCCATGGCCAAGGACATTGAGGTTG |  |
| LcPIP2;3-R(pYES2-LcPIP2;3) | GATGGATATCTGCAGAATTCTTATATTGCGGAAGAGCTCCTGAAGG |  |
| LcPIP2;4-F(pYES2-LcPIP2;4) | TTGGTACCGAGCTCGGATCCATGGCAAAAGACGTTGAAGTAACGG |  |
| LcPIP2;4-R(pYES2-LcPIP2;4) | GATGGATATCTGCAGAATTCTTAGGAATTGCTCCTGAAGGATCCAAG |  |
| LcPIP2;5-F(pYES2-LcPIP2;5) | TTGGTACCGAGCTCGGATCCATGGCAAATGATATAGAAGAAGAGATGGG |  |
| LcPIP2;5-R(pYES2-LcPIP2;5) | GATGGATATCTGCAGAATTCTCACAAACAGGATTGGCTTCTGAATG |  |
| LcPIP2;4-F((pCAMBIA1301-LcPIP2;4-GFP) | AGCTCGGTACCCGGGGATCCATGGCAAAAGACGTTGAAGTAACGG | Gene transient overexpression in fruit and overexpressing in embryonic callus |
| LcPIP2;4-R((pCAMBIA1301-LcPIP2;4-GFP) | TCCTCGCCCTTGCTCACCATGGAATTGCTCCTGAAGGATCCAAG |  |
| GFP-F((pCAMBIA1301-LcPIP2;4-GFP) | GATCCTTCAGGAGCAATTCCATGGTGAGCAAGGGCGAG |  |
| LcMYB306-F((pCAMBIA1301-LcMYB306-GFP) | AGCTCGGTACCCGGGGATCCATGCTCTTGGCTGTCCGTTTT |  |
| LcMYB306-R((pCAMBIA1301-LcMYB306-GFP) | TCCTCGCCCTTGCTCACCATATTCTGAAACAAGCCAGTAGTATCC |  |
| GFP-F((pCAMBIA1301-LcMYB306-GFP) | CTACTGGCTTGTTTCAGAATATGGTGAGCAAGGGCGAGG |  |
| GFP-R((pCAMBIA1301-GFP) | CATGGAAGATCTTCGTCGACTTACTTGTACAGCTCGTCCATGCC |  |
| LcPIP2;4-sg1-F (VK005-14-LcPIP2;4-sg1) | ttgTGCTCCATTGTTCGACGCTG | CRISPR/Cas9 knockout in embryonic callus |
| LcPIP2;4-sg1-R (VK005-14-LcPIP2;4-sg1) | aacCAGCGTCGAACAATGGAGCA |  |
| LcPIP2;4-sg2-F (VK005-14-LcPIP2;4-sg2) | ttgTATCCTCCTTGGAGCTCGT |  |
| LcPIP2;4-sg2-R (VK005-14-LcPIP2;4-sg2) | aacACGAGCTCCAAGGAGGATA |  |
| LcMYB306-sg1-F(VK005-14-LcMYB306-sg2) | ttgTACCGCGCTTAATACCAGGC |  |
| LcMYB306-sg1-R(VK005-14-LcMYB306-sg2) | aacGCCTGGTATTAAGCGCGGTA |  |
| LcMYB306-sg2-F(VK005-14-LcMYB306-sg2) | ttgCGCAAGTCTCCAAGGGTCAG |  |
| LcMYB306-sg2-R(VK005-14-LcMYB306-sg2) | aacCTGACCCTTGGAGACTTGCG |  |
| LcPIP2;4 pro-F(pHIS2-LcPIP2;4 Pro) | ACTCACTATAGGGCGAATTCCCGGTTGAAAATTCCATTCAATCT | Yeast one-hybrid |
| LcPIP2;4 pro-R(pHIS2-LcPIP2;4 Pro) | ATTCGCGAACGCGTGAGCTCTGTTTCCCTGCAAGTAGAGAACAAG |  |
| LcbHLH82-F(pGADT7-LcbHLH82) | CCATGGAGGCCAGTGAATTCATGGATGAGTATTTTGATCAAT |  |
| LcbHLH82-R(pGADT7-LcbHLH82) | TCGAGCTCGATGGATCCTCACGTTTTGGGTTGTCTGG |  |
| LcbHLH107-F(pGADT7-LcbHLH107) | CCATGGAGGCCAGTGAATTCATGGATCCTTCTATTTGGACCTC |  |
| LcbHLH107-R(pGADT7-LcbHLH107) | TCGAGCTCGATGGATCCTCAAGATGACAAGGACGAATCAAACA |  |
| LcSRM1-F(pGADT7-LcSRM1) | CCATGGAGGCCAGTGAATTCATGAACGAAAAAAGCAATTCCTG |  |
| LcSRM1-R(pGADT7-LcSRM1) | TCGAGCTCGATGGATCCTCATTGCATCGGAAATCCAAAACC |  |
| LcMYB4-F(pGADT7-LcMYB4) | CCATGGAGGCCAGTGAATTCATGGGGAGGTCTCCATGTTG |  |
| LcMYB4-R(pGADT7-LcMYB4) | TCGAGCTCGATGGATCCTCAACAATGAAACTGGTGGTGCAG |  |
| LcMYB60-F(pGADT7-LcMYB60) | CCATGGAGGCCAGTGAATTCATGGGAAGGCCTCCTTGC |  |
| LcMYB60-R(pGADT7-LcMYB60) | CTCGAGCTCGATGGATCCTCAGAATATTGGAGAGAGTTCCATCATGTGAT |  |
| LcODO1-F(pGADT7-LcODO1) | CCATGGAGGCCAGTGAATTCATGGGCAGGCAACCTTGT |  |
| LcODO1-R(pGADT7-LcODO1) | TCGAGCTCGATGGATCCTCACAACTTGCCTTCCACGTCTAAAGC |  |
| LcMYB77-F(pGADT7-LcMYB77) | CCATGGAGGCCAGTGAATTCATGGAATGCCCTAGCTCAAGG |  |
| LcMYB77-R(pGADT7-LcMYB77) | TCGAGCTCGATGGATCCTCAAAATCTAATTGCACAGGCAATTGCA |  |
| LcMYB308-F(pGADT7-LcMYB308) | CCATGGAGGCCAGTGAATTCATGTGTCTAGCATCGAAAACAACG |  |
| LcMYB308-R(pGADT7-LcMYB308) | TCGAGCTCGATGGATCCTCAAGGTCTTGGCATGTTGCTTAG |  |
| LcMYB306-F(pGADT7-LcMYB306) | CCATGGAGGCCAGTGAATTCATGCTCTTGGCTGTCCGT |  |
| LcMYB306-R(pGADT7-LcMYB306) | TCGAGCTCGATGGATCCTCAATTCTGAAACAAGCCAGTAGTATCCTC |  |
| LcDIV2-F(pGADT7-LcDIV2) | CCATGGAGGCCAGTGAATTCATGAAGTGGGAAACTGAAGTGATCT |  |
| LcDIV2-R(pGADT7-LcDIV2) | TCGAGCTCGATGGATCCTCATCCATGAGGGTATTGCATCTGAAAAG |  |
| proLcPIP2;4-F(pGreen-0800) | TTGATATCGAATTCCTGCAGCCGGTTGAAAATTCCATTCAATCT | Dual-luciferase reporter assay |
| proLcPIP2;4pro-R(pGreen-0800) | GCGGCCGCTCTAGAACTAGTTGTTTCCCTGCAAGTAGAGAAC |  |
| LcMYB306-F(pCAMBIA1300-LcMYB306) | TCCGCAGCTCCAAAGCTAGCATGCTCTTGGCTGTCCGTTT |  |
| LcMYB306-R(pCAMBIA1300-LcMYB306) | TCGAGCTTGCATGCCTGCAGATTCTGAAACAAGCCAGTAGTATCCTC |  |
| proPIP2;4-F1 | ATAATCAAAGGATATGGACGGCTA | Chromatin immunoprecipitation (ChIP) analysis |
| proPIP2;4-R1 | TGTTTCCCTGCAAGTAGAGAA |  |
| proPIP2;4-F2 | ACCGTGTGTGTATGTGACT |  |
| proPIP2;4-R2 | TAGCCGTCCATATCCTTTGATTAT |  |
| proPIP2;4-F3 | GTTGCTCCAATTGTATTGTAAAGTC |  |
| proPIP2;4-R3 | AGTCACATACACACACGGT |  |
| proPIP2;4-F4 | GGATTAACAGATTATACAGTGCCAAC |  |
| proPIP2;4-R4 | GACTTTACAATACAATTGGAGCAAC |  |
| proPIP2;4-F5 | CATTTCACCCGTCCAAAAGT |  |
| proPIP2;4-R5 | GTTGGCACTGTATAATCTGTTAATCC |  |
| proPIP2;4-F6 | CATGGACTTCTTTAGGCCACA |  |
| proPIP2;4-R6 | ACTTTTGGACGGGTGAAATG |  |
| proPIP2;4-F7 | CCAATAATTTGAACCCTATGGCA |  |
| proPIP2;4-R7 | TGTGGCCTAAAGAAGTCCATG |  |
| proPIP2;4-F8 | GCTGTCATTACCCAGCAATATC |  |
| proPIP2;4-R8 | TGCCATAGGGTTCAAATTATTGG |  |
| proPIP2;4-F9 | TACAGCAGCGTTACCAGG |  |
| proPIP2;4-R9 | GATATTGCTGGGTAATGACAGC |  |
| proPIP2;4-F10 | CCCGTAACAGCTTCATCATC |  |
| proPIP2;4-R10 | CCTGGTAACGCTGCTGTA |  |
| proPIP2;4-F11 | CCGGTTGAAAATTCCATTCAATC |  |
| proPIP2;4-R11 | GATGATGAAGCTGTTACGGG |  |
| proLcPIP2;4-F(Hot/Mutant probe) | Alexa680-CATGGACTTCTTTAGGCCACA | Electrophoretic mobility shift assay |
| proLcPIP2;4-R(Hot probe) | TCTTATCTCTCACTGAAATTTGGTTA |  |
| proLcPIP2;4-R(Mutant probe) | TCTTATCTCTCACTGAAATT**AC**G**AA**A |  |
| LcMYB306-F(pTac-MBP-LcMYB306) | TGTTCCAGGGGCCCGAATTCATGCTCTTGGCTGTCCG |  |
| LcMYB306-R(pTac-MBP-LcMYB306) | AGTCTAGAGTCGACAAGCTTTTAATTCTGAAACAAGCCAGTAGTATCC |  |

**Table S2 LcPIPs in the 'Feizixiao' litchi genome**

| Gene ID | Gene name | Chromosome | start | end |
| --- | --- | --- | --- | --- |
| LITCHI018243.m1 | LcPIP1;1 | Chr15 | 4032890 | 4035096 |
| LITCHI022094.m1 | LcPIP1;2 | Chr10 | 3631128 | 3634829 |
| LITCHI027537.m1 | LcPIP1;3 | Chr3 | 24795425 | 24799029 |
| LITCHI025728.m1 | LcPIP1;4 | Chr3 | 3561070 | 3563830 |
| LITCHI027285.m1 | LcPIP2;1 | Chr3 | 21569205 | 21572878 |
| LITCHI002040.m1 | LcPIP2;2 | Chr5 | 32876569 | 32880418 |
| LITCHI018583.m1 | LcPIP2;3 | Chr15 | 7065928 | 7067754 |
| LITCHI024675.m1 | LcPIP2;4 | Chr13 | 18905743 | 18910277 |
| LITCHI027284.m1 | LcPIP2;5 | Chr3 | 21535739 | 21538785 |

**Table S3 TFs in blue moudule and turquoise module**

|  | Gene ID | TF-Family | Gene name |  | Gene ID | TF-Family | Gene name |
| --- | --- | --- | --- | --- | --- | --- | --- |
| 1 | LITCHI017366.m1 | Alfin-like | AL5 | 26 | LITCHI002226.m1 | FAR1 | FRS5 |
| 2 | LITCHI020245.m1 | AP2/ERF-ERF | ERF114 | 27 | LITCHI027029.m1 | FAR1 | FRS3 |
| 3 | LITCHI025370.m1 | AP2/ERF-ERF | ERF13 | 28 | LITCHI025979.m1 | GARP-G2-like | PSR1 |
| 4 | LITCHI008990.m1 | AP3/ERF-ERF | ERF2 | 29 | LITCHI011609.m1 | GRAS | SCL21 |
| 5 | LITCHI005481.m1 | AP2/ERF-RAV | RAV2 | 30 | LITCHI021190.m1 | GRAS | SCL32 |
| 6 | LITCHI000064.m1 | B3 | - | 31 | LITCHI022755.m2 | HB-HD-ZIP | HAT4 |
| 7 | LITCHI001011.m1 | B3 | ARF1 | 32 | LITCHI016932.m1 | HB-KNOX | KN0X3 |
| 8 | LITCHI011048.m1 | B3 | - | 33 | LITCHI022317.m1 | LIM | WLIM1 |
| 9 | LITCHI001717.m1 | B3 | VRN1 | **34** | **LITCHI024426.m1** | **MYB** | **SRM1** |
| 10 | **LITCHI017467.m1** | **bHLH** | **bHLH82** | **35** | **LITCHI002020.m1** | **MYB** | **MYB4** |
| 11 | **LITCHI020939.m1** | **bHLH** | **bHLH108** | **36** | **LITCHI003293.m1** | **MYB** | **MYB60** |
| 12 | **LITCHI027020.m1** | **bZIP** | **ABI5** | **37** | **LITCHI009134.m1** | **MYB** | **ODO1** |
| 13 | LITCHI023190.m1 | C2C2-CO-like | COL16 | **38** | **LITCHI011086.m1** | **MYB** | **MYB77** |
| 14 | LITCHI017192.m1 | C2C2-GATA | GATA12 | **39** | **LITCHI018253.m1** | **MYB** | **DIV1** |
| 15 | LITCHI028424.m1 | C2H2 | SUF4 | **40** | **LITCHI023106.m1** | **MYB** | **MYB308** |
| 16 | LITCHI004379.m1 | C2H2 | WIP4 | **41** | **LITCHI023435.m1** | **MYB** | **MYB306** |
| 17 | LITCHI008959.m1 | C2H2 | WIP2 | **42** | **LITCHI025061.m1** | **MYB** | **DIV2** |
| 18 | LITCHI016366.m1 | C2H2 | ZNF2 | 43 | LITCHI007894.m1 | NAC | ONAC010 |
| 19 | LITCHI022551.m1 | C2H2 | REF6 | 44 | LITCHI020284.m1 | NAC | NAC83 |
| 20 | LITCHI009809.m1 | C3H | ZC3H37 | 45 | LITCHI020398.m1 | NAC | NAC71 |
| 21 | LITCHI020440.m1 | C3H | ZC3H12 | 46 | LITCHI017567.m1 | NF-YA | NFYA3 |
| 22 | LITCHI028727.m1 | C3H | ZC3H62 | 47 | LITCHI017369.m1 | RWP-RK | NLP1 |
| 23 | LITCHI028728.m1 | C3H | ZC3H62 | 48 | LITCHI004652.m1 | SPL | SPL2 |
| 24 | LITCHI028739.m1 | C3H | ZC3H48 | 49 | LITCHI016098.m1 | WRKY | WRKY32 |
| 25 | LITCHI020487.m1 | E2F-DP | E2FF | 50 | LITCHI019292.m1 | TCP | TCP1 |

**Table S4. The *cis*-acting elements in the PIP2;4 promoter**

| Name of element | Sequence | Number |
| --- | --- | --- |
| A-box | CCGTCC | 2 |
| AAGAA-motif | gGTAAAGAAA | 1 |
| ABRE | GCAACGTGTC、ACGTG | 2 |
| ARE | AAACCA | 2 |
| AT~TATA-box | TATATA | 14 |
| Box4 | ATTAAT | 4 |
| CAAT-box | CAAT、CAAAT、CCAAT、TGCCAAC、 | 46 |
| CCGTCC motif | CCGTCC | 2 |
| ERE | ATTTTAAA | 3 |
| G-Box | CACGTT | 1 |
| GATA-motif | AAGATAAGATT、AAGGATAAGG | 2 |
| GT1-motif | GGTTAA、GGTTAAT | 2 |
| STRE | AGGGG | 1 |
| TATA-box | TATAAAAT、TATA、ATTATA、ATATAT、TATATA、TATAA、TATAAA、TATAAAT、ATATAA、TATAAATA、TATAAAA、TACAAAA、TAAAGATT、taTATAAAtc、TATTTAAA | 96 |
| TC-rich repeats | GTTTTCTTAC | 1 |
| MYB | CAACCA、TAACCA、TAACTG | 7 |
| MYC | CAATTG | 2 |
